# Trm9-Catalyzed tRNA Modifications Regulate Global Protein Expression by Codon-Biased Translation

**DOI:** 10.1371/journal.pgen.1005706

**Published:** 2015-12-15

**Authors:** Wenjun Deng, I. Ramesh Babu, Dan Su, Shanye Yin, Thomas J. Begley, Peter C. Dedon

**Affiliations:** 1 Department of Biological Engineering, Massachusetts Institute of Technology, Cambridge, Massachusetts, United States of America; 2 Department of Cell Biology, Harvard Medical School, Boston, Massachusetts, United States of America; 3 SUNY College of Nanoscale Science and Engineering, Albany, New York, United States of America; 4 RNA Institute and Cancer Research Center, University at Albany, State University of New York, Albany, New York, United States of America; 5 Singapore-MIT Alliance for Research and Technology, Singapore; National Institute of Child Health and Human Development, National Institutes of Health, UNITED STATES

## Abstract

Post-transcriptional modifications of transfer RNAs (tRNAs) have long been recognized to play crucial roles in regulating the rate and fidelity of translation. However, the extent to which they determine global protein production remains poorly understood. Here we use quantitative proteomics to show a direct link between wobble uridine 5-methoxycarbonylmethyl (mcm^5^) and 5-methoxy-carbonyl-methyl-2-thio (mcm^5^s^2^) modifications catalyzed by tRNA methyltransferase 9 (Trm9) in tRNA^Arg(UCU)^ and tRNA^Glu(UUC)^ and selective translation of proteins from genes enriched with their cognate codons. Controlling for bias in protein expression and alternations in mRNA expression, we find that loss of Trm9 selectively impairs expression of proteins from genes enriched with AGA and GAA codons under both normal and stress conditions. Moreover, we show that AGA and GAA codons occur with high frequency in clusters along the transcripts, which may play a role in modulating translation. Consistent with these results, proteins subject to enhanced ribosome pausing in yeast lacking mcm^5^U and mcm^5^s^2^U are more likely to be down-regulated and contain a larger number of AGA/GAA clusters. Together, these results suggest that Trm9-catalyzed tRNA modifications play a significant role in regulating protein expression within the cell.

## Author Summary

Here we present evidence for a more complicated role for transfer RNAs (tRNAs) than as mere adapters that link the genetic code in messenger RNA (mRNA) to the amino acid sequence of a protein during translation. tRNAs have long been known to be modified with dozens of different chemical structures other than the 4 canonical ribonucleosides, though the role of these modifications in controlling translation is poorly understood. By quantifying the expression of thousands of proteins in the yeast *S*. *cerevisiae*, we identified a mechanistic link between modified ribonucleosides located at the wobble position of two tRNAs, tRNA^Arg(UCU)^ and tRNA^Glu(UUC)^, and the translation of proteins derived from genes enriched with codons read by these tRNAs: AGA and GAA. In cells lacking the enzyme that inserts these modifications, tRNA methyltransferase 9 (Trm9), we found a significant reduction in proteins from genes enriched with AGA and GAA codons and with runs of these codons. Also, mRNAs enriched with runs of AGA and GAA codons are subject to stalled translation on ribosomes in yeast lacking mcm^5^U and mcm^5^s^2^U. Together, these results reveal a distinct role for Trm9-catalyzed tRNA modifications in selectively regulating the expression of proteins enriched with AGA and GAA codons.

## Introduction

A striking feature of tRNA molecules is the large number of post-transcriptional modifications, representing up to 10% of the ribonucleoside content [1,2]. Ranging from simple base methylation to complex modifications involving multiple enzymatic steps, modified ribonucleosides are phylogenetically widespread and have long been recognized to play crucial roles in tRNA functions [1,3–5]. Modifications in or around the anticodon loop of tRNA affect translation rate and fidelity through stabilization of codon-anticodon pairing, while other modifications remote from the anticodon loop have specific roles in regulating tRNA stability and folding [1,3,4,6–10]. These observations fuel the hypothesis that tRNA modifications play a broader role in regulating global protein expression, with a focus here on wobble uridine modifications catalyzed by tRNA methyltransferase 9 (Trm9) in budding yeast.

**Fig 1:**
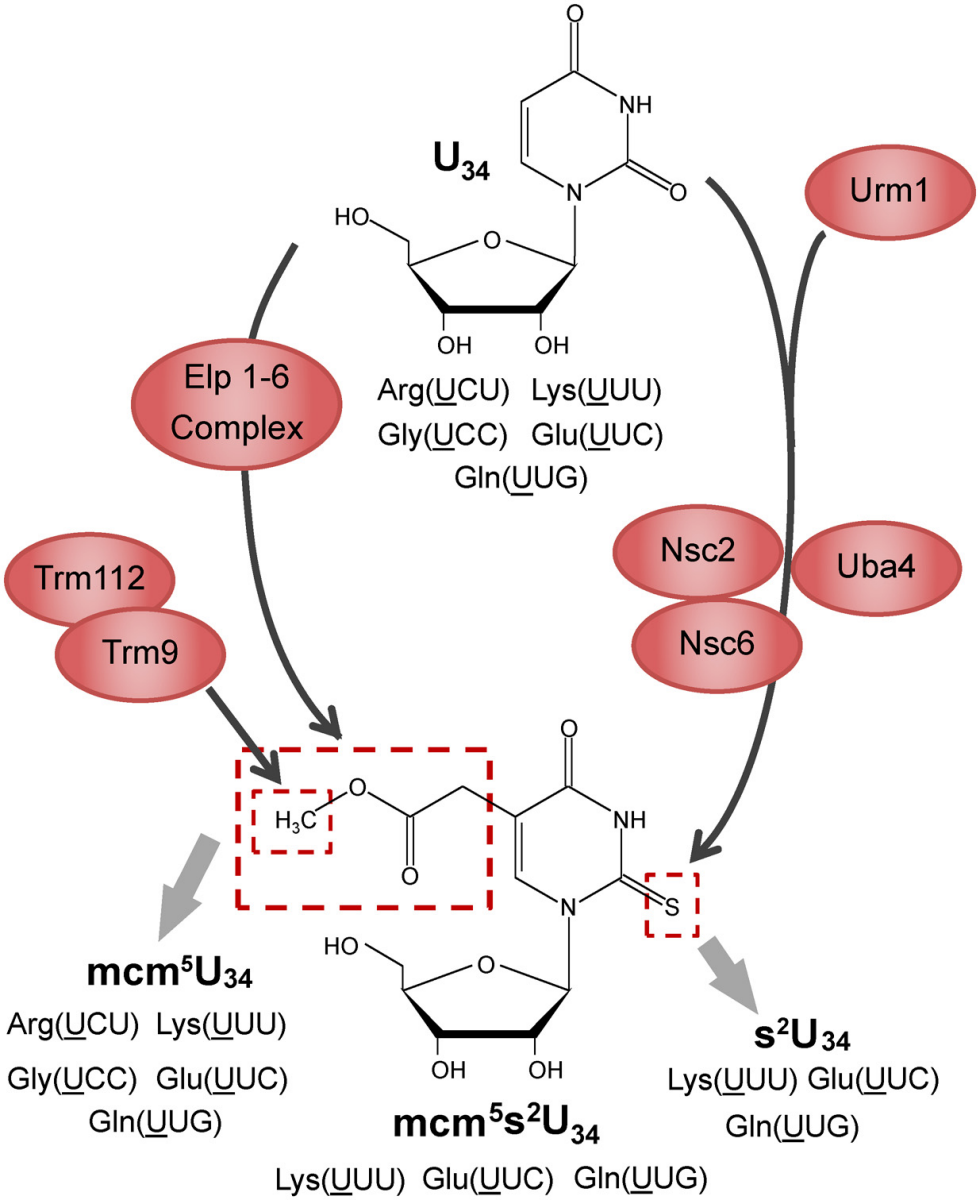
Pathways for the synthesis of modified wobble uridines in tRNA substrates for Trm9 and other enzymes. Synthesis of 2- and 4-thiouridine can precede C5-modification in mitochrondrial tRNAs.

Modification of the wobble uridine in tRNA^Arg(UCU)^, tRNA^Gly(UCC)^, tRNA^Lys(UUU)^, tRNA^Gln(UUG)^ and tRNA^Glu(UUC)^ requires a number of key activities (Fig 1). The Elongator complex (ELP1-ELP6) uses uridine as a substrate and catalyzes the formation of 5-carboxymethyluridine (cm^5^U). In association with Trm112, Trm9 will use cm^5^U as a substrate and catalyze the formation of 5-methoxycarbonyl-methyluridine (mcm^5^U) at the wobble position of tRNA^Arg(UCU)^, tRNA^Gly(UCC)^, tRNA^Lys(UUU)^, tRNA^Gln(UUG)^ and tRNA^Glu(UUC)^ (Fig 1) [11–14]. The wobble position of tRNA^Lys(UUU)^, tRNA^Gln(UUG)^ and tRNA^Glu(UUC)^ is further thiolated by an enzyme cascade involving Urm1, Uba4, Ctu1, Ncs2 and Ncs6 to yield mcm^5^s^2^U (Fig 1) [11,12,14].

We and others have performed studies in budding yeast, which suggest that stress-induced reprogramming of wobble modifications in tRNA leads to enhanced translation of codon-biased mRNAs for critical stress response genes. [15–19]. For example, we showed that deficiencies in Trm9 and another anticodon loop tRNA methyltransferase, Trm4, cause sensitivity to DNA alkylating agents (methylmethane sulfonate, MMS) and reactive oxygen species (H_2_O_2_), respectively [16,20–23]. The roles of the tRNA modifications catalyzed by Trm4 and Trm9 in the cell response were found to involve stress-induced increases in wobble m^5^C in tRNA^Leu(CAA)^ and wobble mcm^5^U in tRNA^Arg(UCU)^ and tRNA^Gln(UUG)^, respectively. With Trm9, these changes were directly linked, by Western blots, to enhanced translation of several stress response proteins enriched with AGA and GAA codons [20]. These results not only support the idea that dynamic changes in tRNA wobble modifications facilitate translation of critical stress response proteins [19,20], but they also raise the possibility of an alternative or accessory genetic code involving selective use of degenerate codons as an adaptation for translational regulation by tRNA modifications.

Although there are several examples of individual genes supporting this notion [8,10,17,20,24], what is lacking in this model for translational control of stress response is the larger view of the role of tRNA modifications in regulating global protein expression. Regulation of protein expression occurs at a variety of different levels [25–27], for example, by regulating transcription activity, splicing efficiency, and mRNA export and stability [25,28–33]. Moreover, different stages of protein synthesis are also subject to regulation to ensure efficiency and to preserve fidelity [25,26,34]. Among the variety of factors regulating gene expression, very little is known about the role of tRNA modifications as determinants of global protein translation. Recent studies have shown that loss of Ctu1 or ELP3 result in a moderate reduction in the global protein expression [10], while *urm1* and *elp3* knockout impairs translation of proteins with high usage of AAA, CAA, and GAA codons [9]. Using ribosomal footprinting, two recent genome-based studies measured average ribosome occupancy on each codon type in several yeast U34 modification mutants [24,35]. While there were striking discrepancies between the two similar studies, the consistent results suggested that loss of wobble uridine modifications in *ncs2*Δ, *ncs6*Δ, *elp3*Δ and *elp6*Δ mutants leads to changes in ribosome occupancy for some codons associated with tRNA^Arg(UCU)^, tRNA^Gly(UCC)^, tRNA^Lys(UUU)^, tRNA^Gln(UUG)^ and tRNA^Glu(UUC)^. Of particular note, however, is that the effects caused by loss of U34 modifying enzymes on overall ribosome occupancy on each codon type were averaged over the genome and thus did not reflect codon usage patterns in individual genes, which greatly limits their regulatory conclusions. To address these problems and identify gene-specific regulatory rules, we performed an integrated analysis of proteome, transcriptome and gene-specific ribosome footprinting to investigate the role of Trm9-catalyzed tRNA modifications, mcm^5^U and mcm^5^s^2^U, in regulating global protein expression.

## Results

### Loss of Trm9 affects levels of mcm^5^U and mcm^5^s^2^U but not Trm9-dependent tRNA species

With the overall goal of assessing the effects of loss of Trm9 and its products mcm^5^U and mcm^5^s^2^U on global protein translation, we first verified that modified ribonucleosides mcm^5^U and mcm^5^s^2^U were absent in the *trm9Δ* cells (S1A and S1B Fig) while the abundance of the hypomodified tRNA species were not significantly affected (S1C Fig) under both normal and stress conditions. These results corroborate previous studies [4,11,12,16,20,36] and establish the *trm9Δ* cells as a well-controlled model system for analyzing the influence of tRNA modification on global protein expression.

### SILAC proteomics identifies differentially expressed proteins in the absence of Trm9-catalyzed tRNA modifications

We then used a SILAC-based quantitative proteomic analysis to assess global protein expression in unexposed and MMS-exposed wild-type (WT) and *trm9Δ* yeast [37]. Proteins derived from *lys1Δ* yeast grown with [^13^C_6_,^15^N_2_]-L-lysine were used as an internal standard that was added to protein extracts in each sample, with quantitation of proteins relative to this standard accomplished by LC-MS/MS analysis of protein digests [37]. Protein coverage was maximized by extensive peptide fractionation using an off-gel isoelectric focusing system [38]. Using this approach, we achieved high-confidence identification of 2,408 proteins with a false-discovery rate of 0.46% (S1 Table) and good reproducibility for the three biological replicates analyzed for each cell type and treatment condition (S1D and S1E Fig). Interestingly, protein expression patterns showed greater similarity between the same yeast strains under different treatment conditions than between different yeast strains under the same conditions, which indicates that loss of Trm9 has a stronger influence on global protein expression than MMS treatment. Altogether, we identified 231 proteins that were significantly down-regulated and 95 up-regulated proteins in *trm9*Δ cells compared with WT cells during normal growth (p <0.05, Student’s t-test and fold-change >1.2) (S2 Table). We also identified 195 significantly down-regulated proteins and 137 significantly up-regulated proteins in *trm9*Δ cells in response to MMS treatment (S2 Table).

### Proteins enriched with AGA and GAA codons are preferentially down-regulated in *trm9*Δ cells

We first examined whether changes in protein expression are highly selective given the assumption that, after controlling for protein length, proteins with enhanced usage of codons dependent on these wobble modifications are more likely to be down-regulated in *trm9*Δ cells. In this case, we would expect to see genes enriched with mcm^5^U-dependent codons AGA and GGA, as well as mcm^5^s^2^U-dependent codons, CAA, GAA and AAA, to be selectively down-regulated. Moreover, it has been shown that the mcm^5^ side chain facilitates wobble decoding for G-ending codons [4]. Accordingly, proteins enriched with AGG and GGG codons may likewise be affected by loss of mcm^5^U.

**Fig 2:**
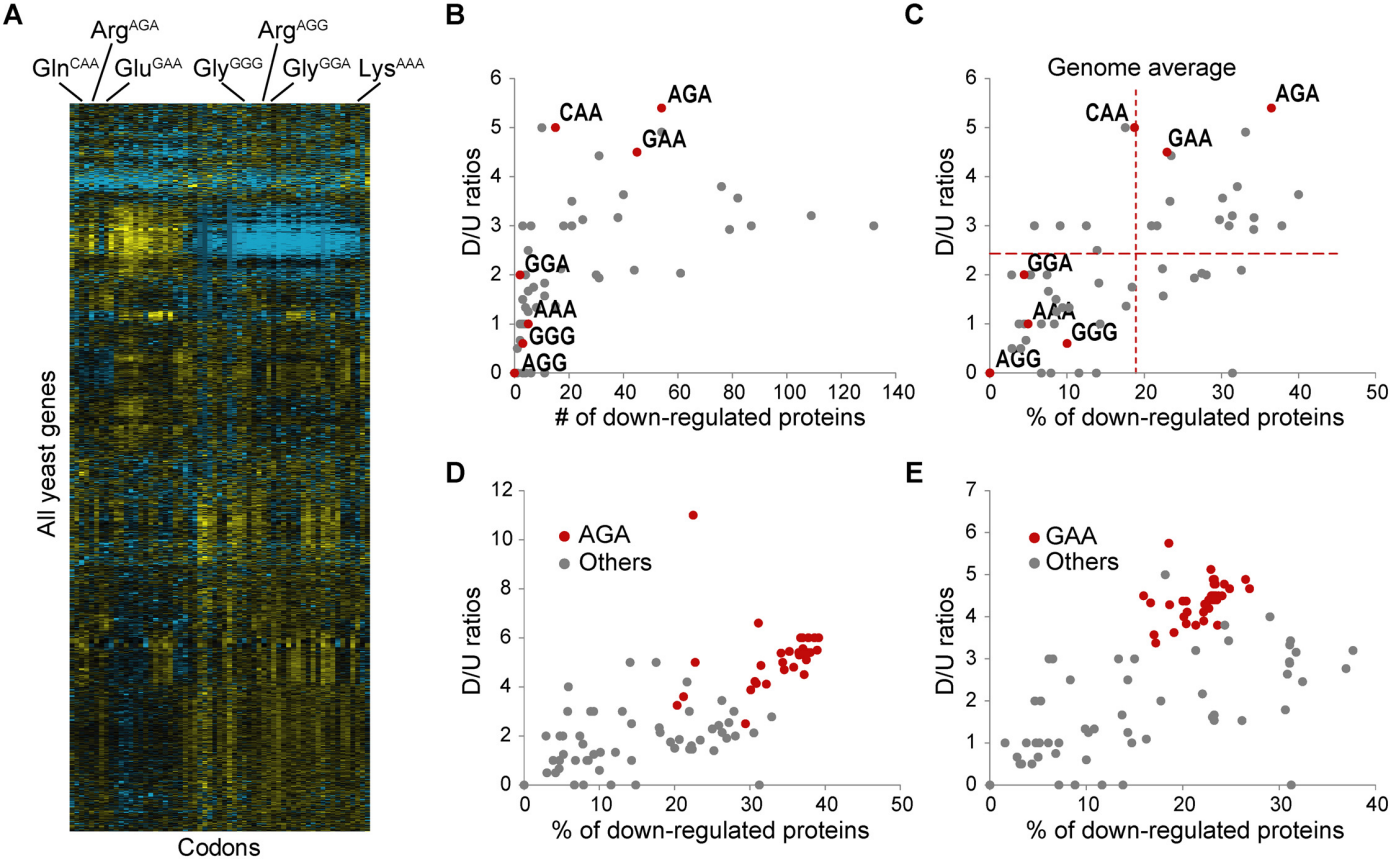
Loss of Trm9 preferentially reduces expression of proteins from genes enriched with AGA and GAA codons, but not other codons. Groups of genes were identified, each with a significantly high use of one of 61 codons. Within each codon group, the number or percentage of significantly altered proteins and the ratio of down- to up-regulated proteins (D/U) were calculated. (**A**) Hierarchical clustering analysis of gene-specific codon usage patterns (Z-scores) for all yeast genes. Z-scores were calculated as the difference between the frequency of each codon used by each transcript and the genome average, divided by the standard deviation, with the scores indicating whether a transcript is over- or under-represented with a specific codon compared to the genome average. Over-represented codons are displayed in yellow and under-represented codons are displayed in blue. Codons showing no difference from the genome average are displayed in black. For clarity dendrograms are not displayed. Data are taken from (**B**) Plot of D/U *versus* the number of significantly down-regulated proteins in each group of proteins enriched with a given codon. The red dots represent data for the groups linked to the codons read by Trm9-modified tRNAs, while grey dots represent data for other codon groups. (**C**) To control for differences in group size, the ratio of down- to up-regulated (D/U) proteins was plotted as a function of the percentage of significantly down-regulated proteins in each group of proteins enriched with a given codon. The red lines indicate the overall percentage of significantly down-regulated proteins and the average D/U ratio in all proteins, respectively. (**D**) To account for codon biases other than AGA, proteins enriched with other codons (except for GAA) were removed from the AGA-enriched group, one codon at a time. Proteins from genes enriched with GAA were likewise removed from groups of proteins enriched in other codons. The values of the remaining proteins enriched with AGA were separately plotted in red dots, while the values of the remaining proteins enriched with other codons were plotted in grey dots. (**E**) Analysis similar to (**D**) repeated for proteins enriched with GAA.

To this end, we analyzed gene-specific codon usage patterns for all 5886 genes in the yeast genome to determine groups of proteins that are significantly enriched with each codon. A Z-score was calculated to indicate whether a certain codon is over- or under-represented in each individual gene compared to the genome average. Hierarchical clustering analysis of Z-scores of all genes revealed clusters of codons with relatively similar patterns of usage across different genes (Fig 2A; codon usage data for individual genes is presented in S3 Table). The heat map in Fig 2A shows clustering of CAA, AGA and GAA codons, which is distinguished from clustering of GGG, AGG and GGA codons, while the AAA codon was separated from the others.

We then asked whether proteins enriched with these wobble modification-dependent codons were selectively down-regulated in *trm9*Δ cells. To this end, we calculated the number of significantly down-regulated proteins in each group of proteins enriched with one of the 61 codons. Moreover, to control for the size of different groups and the randomness of changes in protein expression, the percentage of down-regulated proteins, as well as the ratio of the number of down-regulated proteins to the number of up-regulated proteins (D/U) in each group were calculated. For example, in our proteomic dataset, 148 proteins overrepresented with AGA codon were identified and quantified, of which 54 (36.5%) were significantly down-regulated, while only 10 were significantly up-regulated in *trm9*Δ cells (D/U = 5.4). Among the 196 proteins with high GAA usage, 45 (23.0%) were significantly down-regulated and 10 were significantly up-regulated in *trm9*Δ cells (D/U = 4.5). In contrast, for all 2408 proteins identified, only 231 (9.6%) were down-regulated while 95 were up-regulated (D/U = 2.4). As shown in Fig 2B and 2C, the percentage of down-regulated proteins and D/U ratios were significantly enhanced in AGA- and GAA-enriched groups as compared with the genome average. In addition, the CAA group showed a high D/U ratio but the percentage of down–regulated proteins showed no difference from the genome average. This suggested that proteins from genes enriched with AGA/GAA codons were preferentially down-regulated in *trm9*Δ cells. In contrast, we observed no evidence that expression of proteins from genes enriched with GGA, GGG, AGG or AAA were selectively inhibited in *trm9Δ* cells. One possible explanation for lack of effect is that these codons are all non-optimal codons with low overall usage in the genome (see Discussion).

However, several codons independent of the modifications were also associated with a high proportion of down-regulated proteins, which could be explained by co-enrichment of these codons with AGA and GAA. To investigate the influence of codon co-enrichment, we removed proteins enriched with both AGA and another codon from the group of proteins enriched with AGA, and *vice versa*. As shown in Fig 2D, this analysis revealed that high usage of the AGA codon, to the exclusion of any other codon, remained the single best predictor for protein down-regulation in *trm9Δ* cells. In contrast, as expected, the percentages of down-regulated proteins were reduced after removing proteins whose reduction could be better explained by co-enrichment of AGA codon. A similar result was observed for the GAA enriched group (Fig 2E).

In response to MMS treatment, changes in global protein expression in *trm9Δ* cells were likewise skewed as a function of high usage of AGA and GAA codons, but not the other codons dependent on the wobble modifications (S2A–S2D Fig). Taken together, these results support a role for mcm^5^U and mcm^5^s^2^U modifications in regulating proteins enriched with AGA and GAA codons under both normal growth and stress conditions, establishing that Trm9-catalyzed tRNA modifications play a significant role in regulating protein expression.

### Depletion of mcm^5^U and mcm^5^s^2^U suppresses expression of proteins from genes enriched with AGA and GAA codons in a highly selective manner

**Fig 3:**
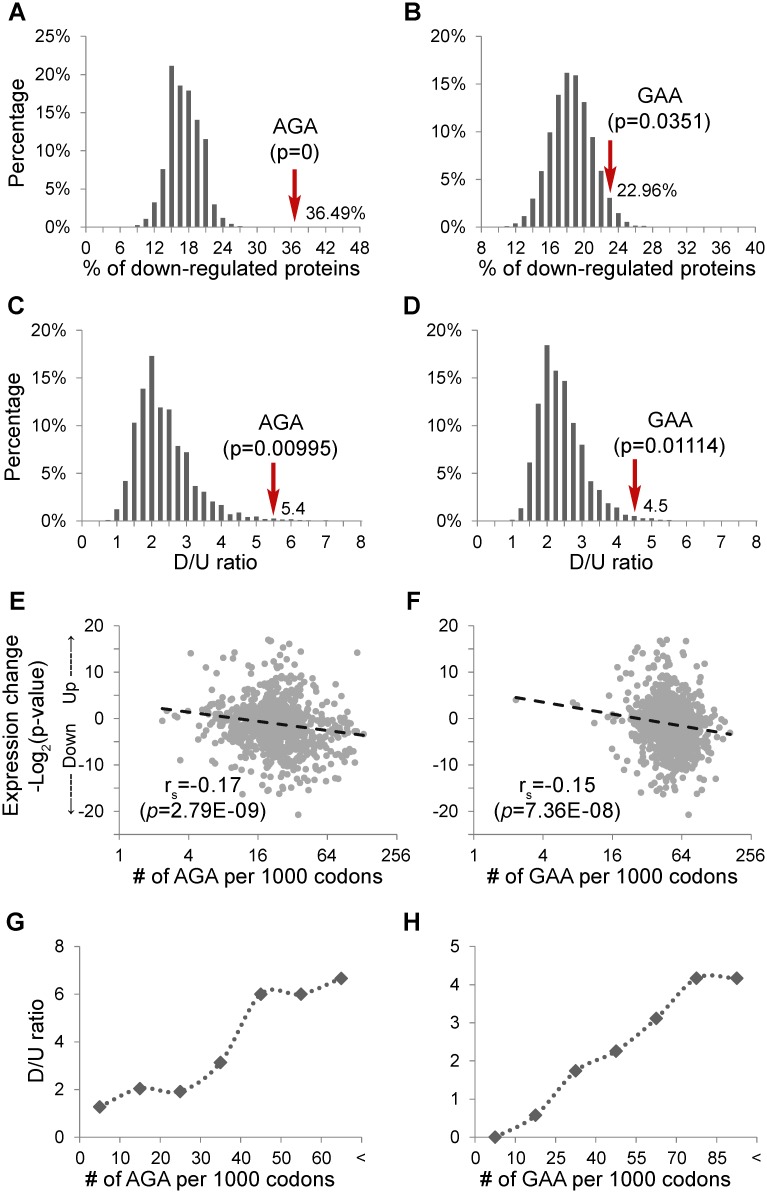
Loss of Trm9 selectively reduces expression of proteins from genes with high usage of AGA and GAA codons. (**A**) Proportion of down-regulated proteins in AGA-enriched proteins *versus* non-enriched proteins. The histogram shows the distribution of proportion of down-regulated proteins of the non-enriched proteins obtained by 100,000 random samplings. The position of AGA-enriched proteins is indicated by an arrow, with the p-values indicating the chance that a random sampling has a ratio no less than that of the AGA-enriched group. (**B**) Proportion of down-regulated proteins of GAA-enriched proteins *versus* non-enriched proteins, as described above. (**C**) The D/U ratio of AGA-enriched proteins *versus* non-enriched proteins. (**D**) The D/U ratio of GAA-enriched proteins *versus* non-enriched proteins. (**E**) Correlation between AGA or (**F**) GAA codon usage and changes in expression of all proteins in *trm9*Δ cells. Spearman rank correlation coefficient (rs) and p-value are shown. (**G, H**) To further assess codon usage among significantly up- and down-regulated proteins, proteins were divided into seven bins based on AGA (**G**) or GAA (**H**) codon frequency (number per 1000 codons; bins of 0–10,10–20, *etc*.), and the average codon frequency in each bin was plotted relative to the ratio of down- to up-regulated proteins (D/U ratio for significantly altered proteins) calculated for each binned group.

We then examined whether proteins enriched with AGA or GAA codons were more likely to be down-regulated than expected by chance in *trm9*Δ cells and whether the results could be better explained by, for example, changes in mRNA level or biased protein expression. To this end, for proteins enriched with AGA codon (n = 148), we performed 100,000 random samplings of 148 proteins from the proteins that are not enriched with AGA codon, and calculated the percentage of down-regulated proteins and D/U ratio for each sampling. This analysis demonstrated that groups of proteins from genes enriched with AGA were more likely to possess a higher proportion of down-regulated proteins (Fig 3A) as well as a greater D/U ratio (Fig 3C) than expected by chance. Similarly, proteins enriched with GAA codons (n = 196) were more likely to be down-regulated than genome average in *trm9Δ* cells (Fig 3B and 3D), but not for those enriched with the other codons dependent on the wobble modifications (S3A–S3J Fig). Taken together, these results suggest that depletion of mcm^5^U and mcm^5^s^2^U represses expression of proteins enriched with AGA and GAA codons in a highly selective manner.

However, this analysis could be misleading without controlling for changes in mRNA levels, which may be a major contributor to the changes in protein expression. To this end, we combined the proteomic data with our previous microarray data from *trm9Δ* cells [19]. We found that changes in protein level and changes in mRNA expression were not correlated, with only 4% (13 out of 326) of the significantly changed proteins explained by changes in mRNA level. We then repeated the analysis after removing these proteins from the dataset. As expected, proteins enriched with AGA and GAA codons were still preferentially down-regulated in *trm9*Δ cells (S4A and S4B Fig).

Another feature in question was a bias caused by protein abundance. As shown in S5A and S5B Fig, proteins from genes with high usage of AGA and GAA codons were skewed toward highly expressed proteins. Accordingly, these proteins may be more dramatically down-regulated because they were present at higher levels in WT cells. To control for this, we repeated the analysis by randomly selecting a group of proteins with the same expression level as proteins enriched with AGA or GAA codon, respectively, in each sampling. As shown in S5C and S5D Fig, after controlling for protein abundance, proteins from genes enriched with AGA or GAA were still more likely to be down-regulated in *trm9Δ* cells than expected by chance.

We then examined the possibility that a specific protein is down-regulated in *trm9Δ* cells as a function of increased usage of AGA/GAA codons in all proteins, regardless of whether they are enriched with AGA/GAA codons or not. As shown in Fig 3E and 3F, we found that reduced protein expression in *trm9Δ* cells was significantly correlated with enhanced usage of AGA (r_s_ = -0.17, p = 2.8E-09) and GAA (r_s_ = -0.17, p = 7.4E-08), respectively. Furthermore, we binned proteins into seven groups based on their usage of AGA and GAA codons and calculated the D/U ratio in each group. As shown in Fig 3G and 3H, increased usage of AGA and GAA codons additively enhanced the chance of down-regulation in *trm9Δ* cells on a genomic scale. These correlations held when we examined the data for MMS-treated cells (S6 Fig). Together, these results establish that depletion of mcm^5^U and mcm^5^s^2^U selectively repressed expression of proteins with high usage of AGA and GAA codons.

### AGA and GAA codons tend to cluster in genes

**Fig 4:**
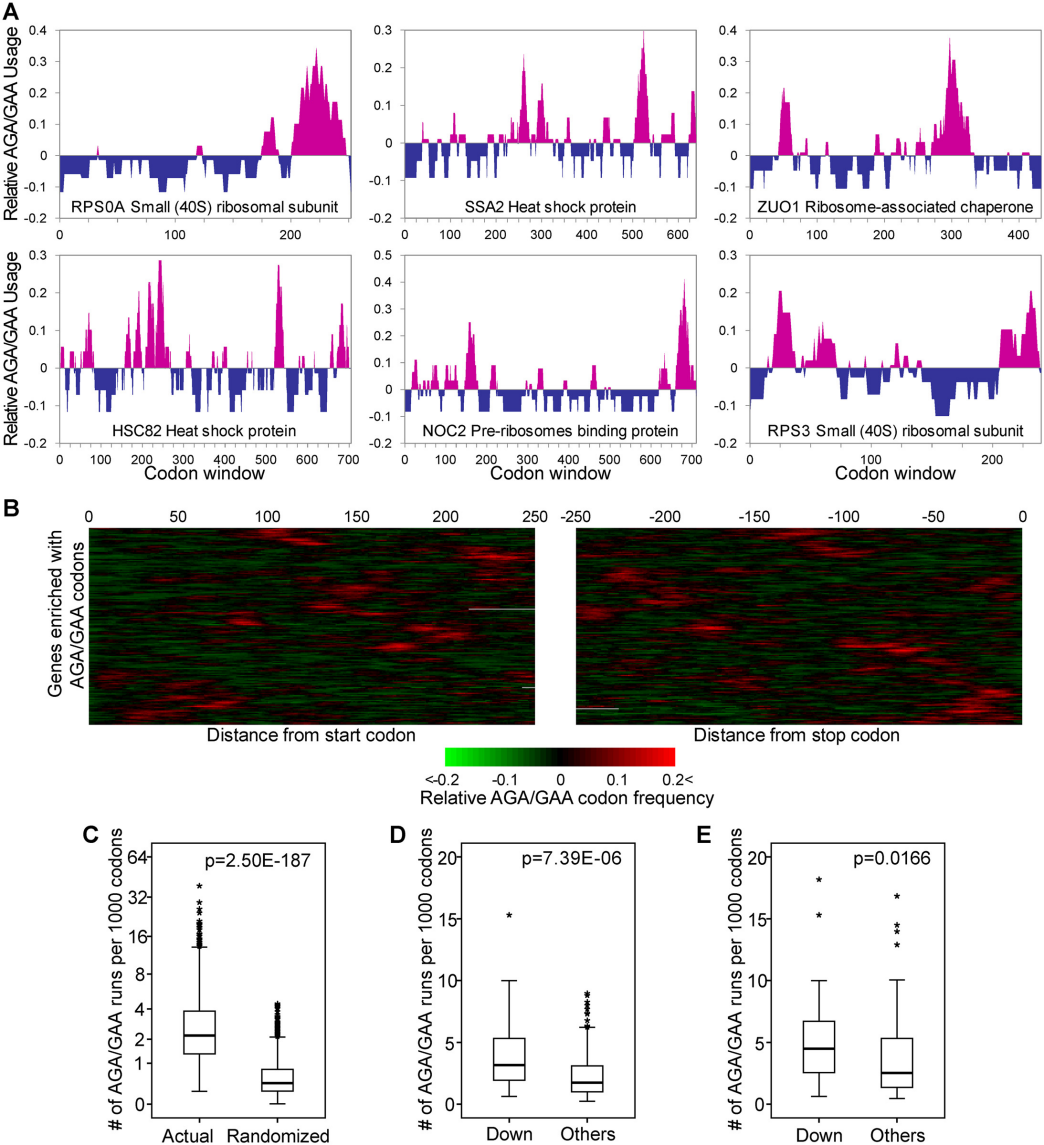
Physical clustering of AGA and GAA codons in genes. (**A**) To illustrate clustering or nonrandom spacing of AGA and GAA codons in several genes, AGA and GAA usage in each gene was calculated in a sliding window size of 15 codons (see Methods) and plotted in the histogram along the length of the gene. Pink peaks correspond to physical clusters of AGA and GAA codons while blue peaks represent regions deficient in these codons. (**B**) The close spacing of AGA and GAA codons is illustrated for all AGA- and GAA-enriched genes in a heat map showing AGA and GAA usage calculated in a sliding window size of 15 codons. For clarity, the map shows regions of genes within 250 codons downstream of the start codon or upstream of the stop codon. (**C**) Boxplot of the number of 3-mer codon runs identified in each gene (actual) and that of shuffled sequences maintaining the codon composition of each gene (randomized). (**D**) Boxplot of the number of 3-mer codon runs identified in significantly down-regulated proteins and in other proteins. (**E**) Similar to (**D**), but only proteins enriched with AGA/GAA codons in both groups were considered. Asterisks in panels C, D and E represent outliers.

In addition to overall codon usage, certain features such as codon clustering (i.e., close spacing of codons along a gene sequence) may also regulate the rate of translation along a transcript. We thus scanned each mRNA sequence with a sliding window searching for physical clustering of AGA and GAA codons. As shown in Fig 4A and 4B, for genes enriched with AGA and GAA codons, we observed non-random distributions of these codons along the transcripts. We then tested whether such clustering occurred more frequently than expected by chance. To this end, we counted the number of triplet runs (3-mer) of AGA and GAA combinations in each gene. Maintaining codon composition, we shuffled the codons of each gene and counted the number of triplet runs. After performing this shuffling 10,000 times for each gene, we found that the actual number of codon runs observed was significantly higher than randomization (Fig 4C, p = 2.5E-187, Mann-Whitney U test). The results remained robust when quadruple or quintuple codon combinations were used (S7A and S7B Fig).

In the absence of mcm^5^U and mcm^5^s^2^U modifications, these codon runs may generate a local sequence unfavorable for translation by enhancing the chance of ribosomal pausing. In line with this notion, we showed that genes for down-regulated proteins in *trm9Δ* cells contained a significantly higher number of AGA and GAA runs than the other proteins (Fig 4D, p = 7.4E-6, Mann-Whitney U test). However, this could be potentially explained if genes for down-regulated proteins contained more AGA and GAA codons, and as a result, a higher number of codon runs. We controlled for this scenario by limiting our analysis to proteins from genes enriched with AGA and GAA codons. We found no significant difference in the usage of these codons between proteins with codon runs (n = 144) and those without codon runs (n = 154) (mean frequency: with = 11.2% *versus* without = 11.0%; p = 0.84, Mann-Whitney U test). Controlling for codon usage, we found that down-regulated proteins still had a significantly higher number of codon runs than the other proteins (Fig 4E, p = 0.017, Mann-Whitney U test). These results support the notion that clustering of certain codons imposes an additional layer of regulation on translation efficiency and provide independent evidence for selective inhibition of proteins from genes enriched with AGA and GAA codons in *trm9Δ* cells.

### Reduced expression of proteins enriched with AGA and GAA codons is linked to enhanced ribosomal pausing in cells lacking mcm^5^U and mcm^5^s^2^U

Ribosome footprinting analysis provides an opportunity to quantify the rate of translation of specific mRNA sequences *in vivo* based on the assumption that the slower a ribosome travels along a specific region of a transcript, the more likely that the ribosomal density in that region will be enhanced. Zinshteyn and Gilbert [24] used ribosome footprinting to assess the effect of mcm^5^U and mcm^5^s^2^U on translation rates in *elp3Δ* yeast cells lacking these modifications and found ribosome accumulations at AAA, CAA, and GAA codons. However, their results were based on genome-average ribosomal occupancy on each codon type, and cannot be used to predict altered expression of individual proteins. We thus integrated this ribosomal footprinting data with our proteomic data to examine whether there is a link between ribosomal pausing and reduced protein expression in cells lacking mcm^5^U and mcm^5^s^2^U.

**Fig 5:**
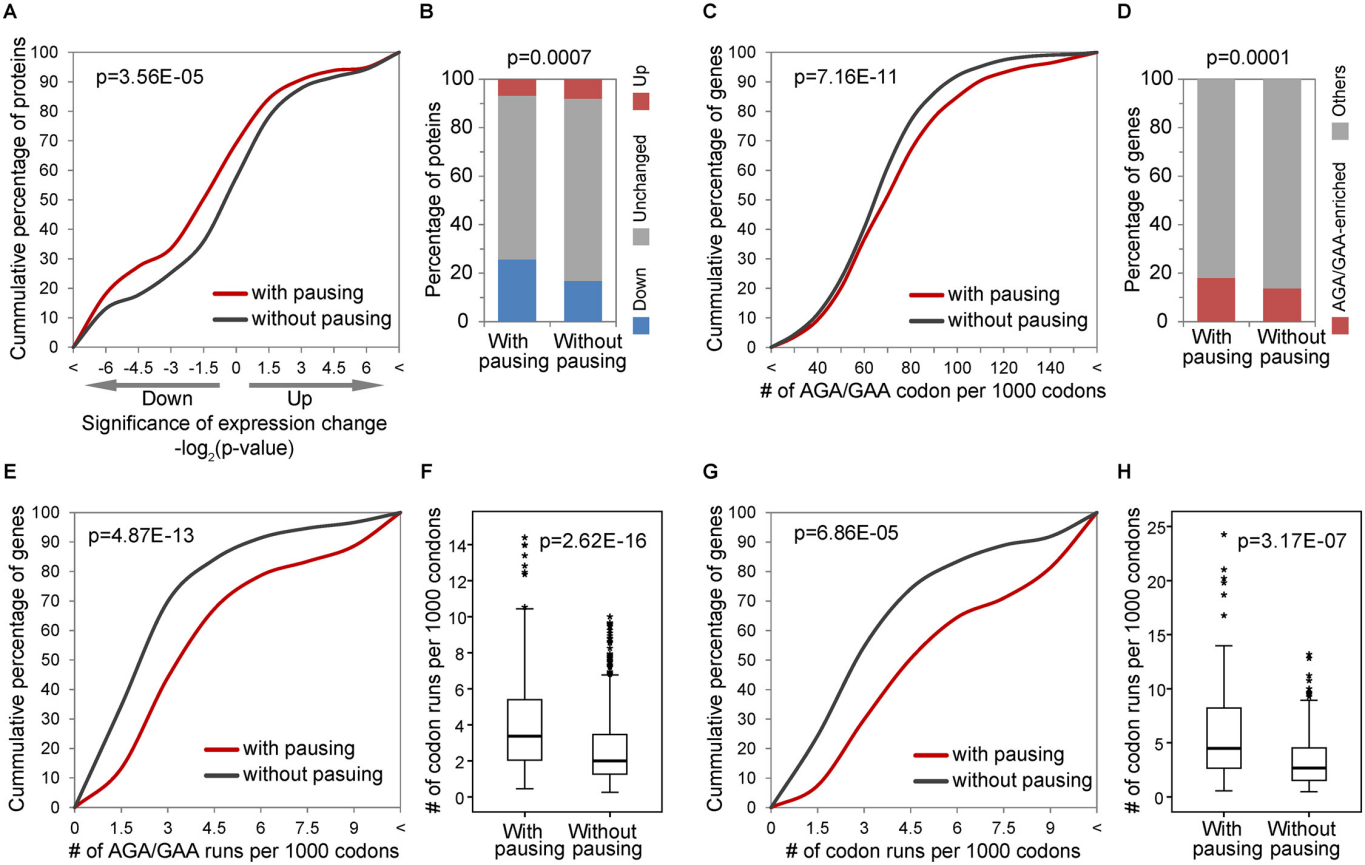
Enhanced ribosomal pausing of proteins enriched with AGA and GAA codons. (**A**) The cumulative percentage of differentially expressed genes representing proteins with or without ribosomal pausing in *trm9*Δ cells. Based on ribosome footprinting data from *elp3Δ* cells lacking mcm^5^U and mcm^5^s^2^U [24] (**B**) Percentages of significantly down-regulated (blue), up-regulated (red) and unchanged (grey) proteins from transcripts with or without ribosomal pausing. (**C**) The cumulative percentage of genes with a given AGA and GAA codon usage, in groups of proteins with or without ribosomal pausing in *trm9*Δ cells. (**D**) Percentages of proteins enriched with AGA and GAA codons (red) and other proteins (grey) in genes with pausing or without ribosome pausing. (**E**) The cumulative percentage of genes with a given frequency of AGA and GAA codon runs, in groups of proteins with or without ribosomal pausing in *trm9*Δ cells. (**F**) Boxplot of the number of codon runs identified in groups of proteins with and without ribosomal pausing. (**G**) Similar to (**E**), but only proteins enriched with AGA and GAA codons in both groups were considered. (**H**) Similar to (**F**), but only proteins enriched with AGA and GAA codons in both groups were considered.

After controlling for differences in sequencing depth and changes in mRNA expression, we calculated the changes in stringently mapped ribosomal densities that occur within a single transcript between *elp3Δ* cells and WT cells [24]. As shown in Fig 5A, proteins whose transcripts have enhanced ribosomal density (pausing) are preferentially down-regulated compared to those without increase in ribosomal density (*i*.*e*., no pausing) (p = 3.56E-05, K-S test). Specifically, as shown in Fig 5B, 75 out of the 292 (26%) transcripts with pausing were found among the significantly down-regulated proteins in *trm9Δ* cells, while only 156 out of the 930 (17%) proteins without pausing were significantly down-regulated in our proteomics dataset (p = 6.92E-4, chi-square test).

We then asked whether ribosomal pausing is associated with enhanced usage of AGA and GAA codons. We note that all genes with ribosome footprinting information were analyzed, regardless of whether they were identified in our proteomic study. As expected, groups of transcripts with pausing tended to contain a higher proportion of genes with high usage of AGA and GAA codons (Fig 5C, p = 7.16E-11, K-S test). Specifically, a significantly higher rate of genes enriched with AGA and GAA codons was observed in genes with pausing (226/1251, 18.1%) than in genes without pausing (598/4353, 13.7%) (Fig 5D, p = 1.39E-4, chi-square test).

We further asked whether clustering of AGA and GAA codons could enhance ribosomal pausing. As shown in Fig 5E, the genes prone to pausing were skewed toward those with more runs of AGA and GAA codons (p = 4.87E-13, K-S test) and the frequency of AGA and GAA codon runs in stalled genes is significantly higher than those without pausing (Fig 5F, p = 2.62E-16, Mann-Whitney U test). However, this bias toward codon runs could simply result from an association of ribosomal pausing with transcripts possessing high AGA and GAA codon usage. To control for this bias, we limited our analysis to proteins enriched with AGA and GAA codons in both groups. As shown in Fig 5G, genes prone to pausing are still skewed toward higher numbers of codon runs (p = 6.86E-05, K-S test) and the number of codon runs is significantly higher in transcripts on stalled ribosomes (Fig 5H, p = 3.17E-6, Mann-Whitney U test). Taken together, these data provide independent evidence that loss of mcm^5^U and mcm^5^s^2^U selectively reduces translation of genes enriched with AGA and GAA codons by causing ribosomal pausing.

### Trm9-dependent proteins are functionally enriched in protein synthesis, cell cycle control and DNA damage response

**Fig 6:**
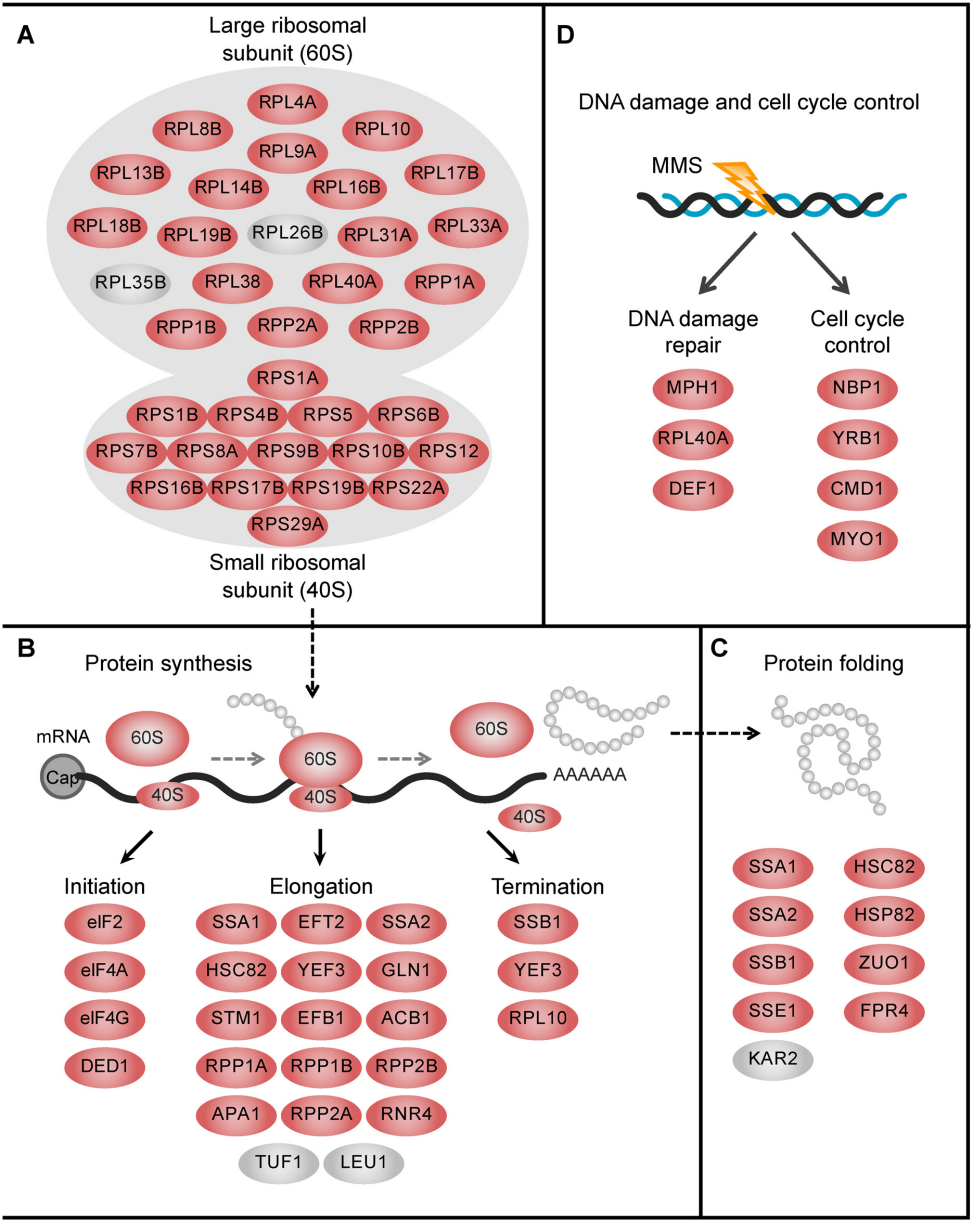
Response to MMS exposure involves Trm9-dependent genes that fall into several tightly connected protein complex and biological processes. (**A**) Ribosomal subunits, (**B**) protein synthesis, including initiation, elongation and termination, (**C**) protein folding and (**D**) DNA damage and cell cycle control. Significantly down-regulated proteins are shown in red and significantly up-regulated proteins are shown in grey. A full list of proteins in different GO contents is provided in S4 and S5 Tables.

The fact that loss of Trm9-catalyzed tRNA modifications disrupts expression of proteins from AGA- and GAA-enriched genes led us to explore the Trm9-dependent proteome for a molecular mechanism underlying the associated phenotype of MMS sensitivity. To this end, we analyzed the biological processes associated Trm9-dependent proteins, with comparison of normally growing and MMS-treated *trm9Δ* cells. Using the David program [36], we find that most of the defects in protein expression occurring in response to MMS exposure are readily observed under normal growth conditions in *trm9Δ* cells (S8 Fig; S4 and S5 Tables). Notably, down-regulated proteins with a unique codon usage pattern linked to Trm9 are heavily enriched in translation machinery. For example, 18 out of the 20 components of the 60S ribosomal subunit and all 15 components of the 40S ribosomal subunit are significantly down-regulated under normal and/or stress condition, indicating impaired function of this basic translation machinery (Fig 6A). Paralleling the reduction in ribosomes, we also observed a down-regulation of proteins involved in different steps of translation (Fig 6B), including eIF2, eIF4A, eIF4g and DED1 involved in translation initiation, 15 out of 17 proteins involved in translational elongation, and SSB1, YEF3 and RPL10 involved in translation termination. Moreover, six out of seven proteins involved in protein folding were significantly down-regulated (Fig 6C). Notably, 11 of the 12 aminoacyl-tRNA synthetases were likewise significantly down-regulated. These results revealed an unexpected role for Trm9-catalyzed tRNA modifications in regulating translation, which is consistent with our previous observation that loss of Trm9 impaired expression of proteins involved in translation elongation (YEF3) and DNA damage repair (RNR1 and RNR3), and leaded to translational infidelity, protein errors and activation of protein stress response pathways [19,20]. This is held up to explain the observation that some proteins without high usage of AGA and GAA codons were also down-regulated in *trm9Δ* cells. However, we also suggest that the effect, if any, could not far surpasses that induced by codon usage bias, otherwise we should not observe the selective repression of proteins enriched with AGA and GAA codons. In addition to translation components, we also observed significant down-regulation of proteins involved in DNA damage repair (MPH1, RPL40A and DEF1) and cell cycle control (NBP1, YRB1, CMD1 and MYO1) pathways (Fig 6D). This is consistent with our previous observations that *trm9Δ* cells display delayed transition into S-phase following DNA damage [19].

## Discussion

The variety and conservation of modified ribonucleosides in tRNA support the idea that they must play an important role in regulating protein expression [2,3,8,17,19], though the evidence remains largely circumstantial without testing their influence on a global scale *in vivo*. To this end, we integrated proteome, transcriptome and ribosome footprinting data to elucidate the role of mcm^5^U and mcm^5^s^2^U in regulating global protein expression. After controlling for various confounding factors, such as protein abundance, changes in mRNA levels, and potential influence of other codons, we found robust evidence that expression of proteins enriched with AGA and GAA codons, and to a lesser extent with CAA, are preferentially repressed in cells lacking mcm^5^U and mcm^5^s^2^U under both normal and stress conditions. Consistent with this result, we previously examined expression of several TAP-tagged endogenous proteins, and found that loss of Trm9 only affected expression of proteins overrepresented with AGA and GAA codons [20]. Moreover, we re-engineered a Trm9-depedent gene, ribonucleotide reductase 1 (RNR1), to replace all Trm9-dependent codons with Trm9-independent synonymous codons. In striking contrast to the wild-type gene, the mutant RNR1 gene was largely resistant to *trm9*Δ-induced repression of protein expression [19]. A combined consideration of the proteomic results presented here and previous genetic studies [39] reveal a highly important role of wobble uridine modifications in regulating global gene expression.

Our data clearly revealed that loss of Trm9 and its wobble modifications causes a significant shift to reduced expression of AGA- and GAA-enriched genes. However, it is important to point out that this regulation is not an “all or none” effect—that is, the loss of Trm9 causes a significant shift in translation but not a complete down-regulation of all AGA- and GAA-enriched genes. The observation that not all AGA- and GAA-enriched genes are affected by loss of Trm9 illustrates the fact that gene expression in general and translation in particular are regulated by a complex interplay of different factors that control the efficiency and fidelity of different steps of protein synthesis. It is also important to point out that we compared groups of proteins enriched or not enriched with a single codon as an unbiased test of the hypothesis that the effects of Trm9 loss should be more pronounced for proteins enriched with Trm9-dependent codons. This proved to be the case, but does not imply that genes enriched with AGA and GAA codons are the only ones affected by loss of Trm9, or that all genes enriched with AGA and GAA must be affected by Trm9 loss. However, we did not see any evidence that genes enriched with other Trm9-dependent codons, including GGA, GGG, AGG and AAA, were preferentially down-regulated in cells lacking Trm9. This could be explained by, for example, lower usage of these codons in yeast genes relative to AGA and GAA codons, or that the effect was counterbalanced by poor usage of AGA, GAA and CAA codons, which is supported by the codon usage clustering result in Fig 2A. Nonetheless, the key point is that our results clearly established, as a proof of concept, that Trm9-dependent tRNA modifications play a significant role in regulating protein expression *in vivo*.

An interesting feature of AGA- and GAA-enriched genes is the observation that the codons are more likely to cluster together than expected by chance. Such clustering has been found to affect local translation rate, which has emerged as a mechanism to fine-tune protein expression and minimize protein folding errors, thus providing an additional layer of translational control. For example, biased combinations of codon runs differ in their propensity to cause mistranslation or ribosome pausing [40–42]. Furthermore, large codon clusters could have a greater effect on protein production than an equivalent number of randomly scattered codons, while clustering of rare codons could play an important role in regulating tissue-specific protein expression [40,43,44]. Here we found that Trm9-dependent proteins from genes enriched with AGA and GAA codons showed a significantly increased frequency of AGA and GAA codon runs. Our results provide evidence that 1) proteins with AGA/GAA codon runs, after controlling for codon usage, are more likely to be down-regulated in *trm9Δ* cells than those without codon runs, and that 2) ribosomal pausing in yeast cells lacking mcm^5^U and mcm^5^s^2^U is more likely to occur with transcripts possessing a larger number of AGA/GAA codon runs. These results support the idea that codon clusters add another layer of translational control to protein production.

Since our proteomic analyses provide new insights into the functional complexity of wobble uridine modifications in regulating translation, it is important to place our results in the context of published studies that address U34 modifications from other perspectives and reveal a highly complicated system of regulatory control. Here we compare our results for Trm9-dependent modifications with the genetic studies (tRNA over-expression rescue) of Bjork and coworkers in *ncs6*Δ and *elp3*Δ mutants [39], as well as with ribosome footprinting studies of Zinshteyn and Gilbert [24] and, more recently, Nedialkova and Leidel [35] in *ncs2*Δ, *ncs6*Δ, *elp3*Δ and *elp6*Δ mutants. S6 Table summarizes these studies. Most notably, the footprinting data showed that the loss of *ncs2*Δ, *ncs6*Δ and *elp3*Δ caused ribosomal pausing on codons GAA, CAA, AAA and GAG, but not AGA, and the authors speculated that the effect was too small to influence protein production [24]. In addition to discordant conclusions based on the same yeast mutants [24,35] and misinterpretation of oxidative stress affects on U34 modifications [35], we point to several problems with these footprinting studies in terms of the uncertainty of both RNA-seq technology and data analytics, as detailed in a recent review of ribosome profiling technology [45], problems that obviate stringent comparisons of the ribosome footprinting data sets with the proteomic data. In terms of data analytics, the two studies analyzed the RNA-seq footprinting data in terms of genome-average effects of ribosomal pausing on each codon type and did not specify ribosomal pausing on individual genes at single-codon resolution [24,35]. Ribosomal pausing on the same codon can vary dramatically among genes and even along the same transcript, depending upon the structural and physiochemical properties of the local protein sequence. So the approach to data analysis used in the two ribosome footprinting studies precludes drawing conclusions about ribosomal pausing on individual transcripts or changes in expression of individual genes. To address these problems, we reanalyzed the *elp3*Δ ribosome footprinting data of Zinshteyn and Gilbert [24] in terms of individual genes and found a significant association between enhanced ribosomal pausing and high usage of AGA/GAA codons, as well as the number of AGA/GAA runs along the transcripts. Another feature of the footprinting data, which involves a focus on short mRNA fragments protected by a single ribosome, could explain the apparent absence of AGA codons among the codons associated with ribosome pausing. Close spacing of paused ribosomes has been shown to produce longer protected RNA fragments that are ignored in most ribosomal footprinting methods [45]. Such close spacing could occur by strong pausing at a high density of AGA codons, with a possible contribution from the drag produced when positively charged Arg residues (coded by AGA) interact with the negatively charged ribosomal exit tunnel [46]. This could explain why overall ribosomal occupancy on AGA codons actually decreased in *elp6Δ* cells in the studies of Nedialkova and Leidel [35]. Lastly, all mutants used in the footprinting studies have modification deficiencies that result in the presence of wobble U or s^2^U on specific tRNAs, while the *trm9* mutant used here leaves wobble cm^5^U or cm^5^s^2^U and the *nsc6* mutant used in Bjork et al. [39] leaves a wobble mcm^5^U. These different wobble modification structures further confound the comparison of the results of the various studies and highlight the complexity of U34 modification effects.

Our analysis of proteins regulated by mcm^5^U and mcm^5^s^2^U modifications provides several insights into the molecular mechanisms underlying the Trm9-dependent alkylation stress response in budding yeast. First, our proteomic analysis revealed 153 of the proteins found to confer sensitivity to MMS in a phenotypic screening study [22], with 30 of these down-regulated and 7 up-regulated by loss of Trm9 (S7 Table). These proteins may thus play a role in the MMS stress response phenotype. An analysis of the functional categories of proteins down-regulated in *trm9Δ* cells provides insights into the molecular mechanisms. One striking feature is that a majority of down-regulated proteins in *trm9*Δ cells are ribosome related or involved in different steps of translation (Fig 6). This behavior has strong parallels with Trm4-dependent translation of ribosomal protein paralogs from TTG-enriched genes during the response to oxidative stress [17]. As one of the central control points for gene expression, protein synthesis is regulated at multiple levels for translation efficiency and error reduction [19,26], with ribosomal protein variants allowing control of ribosome structure and function for plasticity in the cell response to environmental changes and stress [47]. So it is not surprising that loss of Trm9 causes in large-scale changes in protein expression as a result of perturbations of the translational machinery, even for proteins not enriched with AGA and GAA codons. The additional changes in expression of elongation factors could alter local translation rates, leading to mis-folding and impaired protein function [41], which would be compounded by the down-regulation of chaperone proteins observed in *trm9*Δ cells. Consistent with the idea that cells lacking Trm9 suffer from translational inadequacy, loss of Trm9 was found to perturb polysome profiles of Trm9-dependent transcripts [20], to cause a mis-folded protein stress due to multiple translational errors including mis-incorporations and frame shifts [19], and to disrupt translation of AGA, GAA, CAA, GAG codons [19]. The sum of these dysregulations thus induces accumulation of mis-translated and mis-folded proteins that activates protein stress response pathways in *trm9*Δ cells, which is consistent with the fact that loss of Trm9 recapitulates the stress response associated with exposure to the protein- and nuclei acid-damaging agent, MMS.

Another feature of the *trm9*Δ phenotype involves up-regulation of energy production. Since both protein synthesis and rescue of mis-folded proteins by chaperones are energy-dependent processes (S8 Fig; S4 and S5 Tables) [41], it is likely that energy demands are elevated in *trm9*Δ cells to maintain translation activities and to activate degradation pathways for errors in protein translation and folding. This model is supported by our proteomics data and Gene Ontology analysis. In comparison with WT cells, we find that proteins involved in glucose metabolism and the tricarboxylic acid cycle are coordinately up-regulated in *trm9*Δ cells under both normal and MMS stress conditions (S8 Fig; S4 and S5 Tables), possibly to keep up with the increased requirement for energy consumption due to translational errors caused by loss of Trm9 [19]. Oxidative stress response proteins were also activated in *trm9*Δ cells (S8 Fig; S4 and S5 Tables), suggesting that loss of Trm9 confers a state of oxidative stress with elevated reactive oxygen or nitrogen species that are harmful to the cell.

The observed proteomic changes all reflect a phenotype characterized by cell stress, which is consistent with the up-regulation of proteins involved in apoptosis and cell death in *trm9*Δ cells (S8 Fig). Indeed, loss of Trm9 partially recapitulates the MMS-induced stress response in budding yeast [20]. In the absence of Trm9-catalyzed tRNA modifications, cells experience a dysregulated uncoupling of modified tRNAs from codon-biased translation, which leads to a highly regulated but unfavorable steady-state of altered protein synthesis, energy metabolism and cell death. In this regard, we propose a novel model in which regulation of the spectrum of modified ribonucleoside levels at wobble positions in the system of tRNAs fine-tunes global protein expression by codon-biased translation of mRNAs and reprogramming of translational machinery. Trm9 activity thus illustrates a systems-level mechanism for translational control of cell behavior, with mechanistic parallels in other tRNA modification enzymes.

## Materials and Methods

### Materials

Urea, sodium chloride, Tris, sodium fluoride, β-glycerophosphate, sodium orthovanadate, sodium pyrophosphate, dithiothreitol, iodoacetamide were purchased from Sigma Chemical Co. (St. Louis, MO). Endoproteinase Lys-C was purchased from Wako (Richmond, VA). All chemicals and reagents were of the highest purity available. All strains of *S*. *cerevisiae* BY4741 were purchased from American Type Culture Collections (Manassas, VA).

### Yeast strains and treatment

Yeast strain BY4741 was used in this study. WT and *trm9Δ* yeast cells were grown in yeast nitrogen base (YNB) liquid medium containing 30 mg/l normal L-lysine. MMS at a concentration of 0.0125% was used to treat log-phase yeast cells (OD_600_ 0.7) for 1 h, which caused a lethality of <5%. *lys1Δ* yeast cells were grown in YNB medium containing U-[^13^C_6_,^15^N_2_]-lysine (Sigma) at 30 mg/l for at least 10 generations to log-phase. Cells were harvested by centrifugation for 10 min at 1,500 × g at 4°C and washed twice with cold water.

### Quantification of tRNA modifications

Modified ribonucleosides in cytoplasmic tRNA were identified and quantified as reported previously [16,17]. Briefly, WT and *trm9Δ* yeast cells were lysed by lyticase treatment in the presence of deaminase inhibitors (5 μg/ml coformycin, 50 μg/ml tetrahydrouridine) and antioxidants (0.1 mM desferrioxamine, 0.1 mM butylated hydroxytoluene). Total RNA were extracted by the Trizol-chloroform method following manufacturer's instructions, and tRNA-containing small RNA species were enriched using the PureLink miRNA Isolation Kit (Invitrogen). The quantity of tRNA was then determined by UV-vis spectrophotometer (absorbance at 260 nm) and Bioanalyzer analysis (Agilent Bioanalyzer Small RNA Kit). Using identical quantities of tRNA from WT and *trm9Δ* cells, each tRNA sample was mixed with [^15^N]_5_-2-deoxyadenosine as an internal standard and the tRNA was enzymatically hydrolyzed with nuclease P1 and RNase A, followed by dephosphorylation by alkaline phosphatase [16,17]. Proteins and other large molecules were removed by ultrafiltration, and the digested ribonucleosides were then resolved by reversed-phase HPLC (Agilent 1100) with a mobile phase of 1~100% acetonitrile in 8 mM ammonium acetate at a flow rate of 300 μl/min for 1 h. Eluted ribonucleosides were analyzed on an Agilent 6410 Triple Quadrupole mass spectrometer. Modified ribonucleosides were identified by HPLC retention time and collision-induced dissociation (CID) fragmentation pattern. The signal intensity for each ribonucleoside was normalized with the signal intensity of [^15^N]_5_-dA and abundance of the modified ribonucleosides was compared for WT and *trm9Δ* cells in the presence and absence of MMS.

### Northern-blot analysis of tRNA species

DNA probes complementary to 5sRNA (5'-TGGTAGATATGGCCGCAACC-3'), tRNA^Arg(UCU)^ (5'-CACGGCTTAGAAGGCCGTTG-3'), tRNA^Glu(UUC)^ (5'-CTCCGCTACGGGGAGTCGAAC-3'), tRNA^Gln(UUG)^ (5'- GGTCGTACTGGGAATCGAACCCAGG-3'), tRNA^Lys(UUU)^ (5'-CTCCCACTGCGAGATTCGAACTCGC-3') and tRNA^Gly(UCC)^ (5'-GTGTAGTGGTTATCATCCCACCCTTC-3') were end labeled with T4 polynucleotide kinase (NEB) in the presence of [^32^P]-ATP according to the manufacturer’s instructions. Total RNA (10μg) extracted by the Trizol-chloroform method was separated on a 12% polyacrylamide/8M urea/1×TBE gel followed by semi-dry electroblotting onto Hybond N+ nylon membranes (GE Healthcare). Membranes were cross-linked and pre-hybridized for 1 h in hybridization buffer (50% formamide, 0.5% SDS, 5×SSPE, 5×Denhardt’s solution, and 20 μg/ml sheared, denatured, salmon sperm DNA). Then the membrane was hybridized in the same solution containing 10 pmol radio labeled probes overnight at 42°C. The membrane was washed 3-times with 4×SSC at ambient temperature for 10 min. The membrane was wrapped in plastic wrap (Saran) and placed in a cassette with Kodak MS film at -80°C overnight.

### SILAC-based yeast proteomic analysis

Light SILAC-labeled WT and *trm9Δ* yeast cells as well as heavy-labeled *lys1Δ* yeast cells were disrupted in an alkaline buffer (2 M NaOH, 8% v/v 2-mercaptoethanol), and proteins were isolated by TCA precipitation. Yeast proteins were pelleted by centrifugation for 15 min at 15,000 × g at 4°C, and resuspended in lysis buffer (8 M urea, 75 mM NaCl, 50 mM Tris, pH 8.2, 50 mM NaF, 50 mM β-glycerophosphate, 1 mM sodium orthovanadate, 10 mM sodium pyrophosphate, 1 mM PMSF). Protein concentration was determined using the Bradford assay. This *lys1*Δ yeast protein extract was used as global internal standard and mixed (1:1, w/w) with WT and *trm9*Δ proteins separately.

The protein mixture was reduced for 2.5 h at 37°C in 1 mM dithiothreitol (DTT), alkylated for 40 min by 5.5 mM iodoacetamide (IAA) at ambient temperature in the dark, and then digested with 50:1 (w/w) endoproteinase lys-C overnight at 37°C. Peptide mixtures were fractionated into 24 fractions according to their isoelectric point using Agilent 3100 OFFGEL Fractionator (Agilent). Each peptide fraction was acidified by adding 0.1% formic acid, and loaded onto a C18 trap column (200Å Magic C18 AQ 5μm, 150 μm × 10 mm) at flow rate of 5 μl/min, with subsequent elution from a coupled analytic column (200Å Magic C18 AQ 5 μm, 75 μm × 150 mm) at 200 nl/min using a 2–98% acetonitrile gradient (180 min) in 0.1% formic acid. Eluted peptides were analyzed on a QSTAR-XL (Applied Biosystems/MDS Sciex) mass spectrometer. Three technical replicates were performed for each sample.

Acquired MS/MS spectra were parsed by Spectrum Mill (Agilent) and searched against the Saccharomyces Genome Database (SGD). SILAC peptide and protein quantitation was performed with DEQ (Differential Expression Quantitation). SILAC protein ratios are determined as the average of all peptide ratios assigned to the protein, and the proteins quantified in at least two replicates of the sample are recruited for further study. Differential protein expression was determined by Student’s t-test between different samples.

### Gene-specific codon usage

Using protein-coding sequences for 5886 *Saccharomyces cerevisiae* genes downloaded from the SGD database (http://www.yeastgenome.org/), we applied custom scripts to determine the gene-specific codon frequency in terms of the number of a particular codon per thousand codons in the open reading frame. Whether a gene was over- or under-represented with a specific codon relative to the genome average was determined by calculating a Z-score based on a hypergeometric distribution with a cut-off of p < 0.01. Gene-specific codon usage data were analyzed by hierarchical clustering using Cluster 3.0, and visualized as a heat map using Treeview [48]. Simulation and ORF shuffling were performed in R project using custom scripts. A spreadsheet containing the gene-specific codon usage data is presented in

### AGA and GAA codon cluster

We investigated gene-specific clusters of AGA and GAA by calculating the frequency of AGA/GAA codons over a sliding window which was then subtracted the mean value of that gene. Similar to previous studies [49,50], a window size of 15 nt was used in this study. The data were plotted as a histogram, with positive peaks indicating clusters of AGA and GAA codons in these regions. The number of short codon runs in the form of triplets of AGA, GAA or their combinations in each gene was counted using custom scripts in R project.

### Ribosomal footprinting dataset

Ribosomal footprinting data of the wildtype and *elp3Δ* cells as well as the RNA-seq data of the corresponding samples were obtained from a previous study (GSE45366) [24]. The reads per kilobase per million mapped reads (rpkms) of ribosomal footprinting data (FP) and the matched RNA-seq data (T) were used for comparison after normalization for library size. FP/T ratio for each gene was calculated to indicate ribosomal density on each transcript. A cut-off of >1.2 fold increase in FP/T ratio was used to determine whether ribosomal density on a certain gene was enhanced in cells lacking mcm^5^U and mcm^5^s^2^U.

## Supporting Information

S1 Fig Identification of changes in tRNA modifications and protein expression in *trm9*Δ cells. (**A**) The chromatograms indicate that depletion of Trm9 results in a total loss of the modified ribonucleosides mcm^5^U and mcm^5^s^2^U in yeast cells under normal conditions and (**B**) in response to MMS treatment. Modifications were identified by HPLC retention time and collision-induced dissociation fragmentation patterns. (**C**) Northern-blot analysis of tRNA abundance in wild-type (WT) and *trm9*Δ cells under normal condition and in response to MMS treatment. (**D**) Relative standard deviation (RSD) of SILAC ratios between three replicates of each sample. RSD is the absolute value of the coefficient of variation calculated by dividing the standard deviation by the mean value. The median RSD for SILAC quantification was 0.068. (**E**) Hierarchical clustering of SILAC ratios (which indicate relative protein abundance) of all proteins identified in replicates of WT and *trm9*Δ cells under normal and stress conditions.
(JPG)

S2 Fig Proteins enriched with AGA and GAA codons are preferentially down-regulated in *trm9*Δ cells in response to MMS treatment. (**A**) Plot of D/U ratio versus the number of significantly down-regulated proteins in each group of proteins enriched with one certain codon is shown. The red dots represent codons dependent on the wobble modifications, while grey dots represent data of other codon groups. (**B**) Plot of D/U ratio versus the percentage of significantly down-regulated proteins in each group. The red line indicates the percentage of significantly down-regulated proteins in total proteins. (**C**) To control for biased usage of codons other than AGA (except for GAA), proteins enriched with other codons were removed from the AGA-enriched group, one at a time. Proteins enriched with AGA were likewise removed from other codon groups. The values of the proteins enriched with AGA to the exclusion of other codons were separately plotted in red dots, while the values of the proteins enriched with other codons, after removing AGA-enriched proteins, were plotted in grey dots. (**D**) Analysis similar to (**C**) repeated for proteins enriched with GAA codon.
(JPG)

S3 Fig Depletion of mcm^5^ and mcm^5^s^2^ did not selectively repress expression of proteins enriched with other codons. (**A**) The percentage of down-regulated proteins of CAA-enriched proteins *vs* non-enriched proteins. The histogram shows the distribution of the percentages obtained by 100,000 random samplings of the non-enriched proteins. The position of CAA-enriched proteins to that distribution is indicated by arrow, with the p-values indicating the chance that a random sampling has a value no less than that of the CAA-enriched group. Similar results were observed for (**B**) GGA-, (**C**) AAA-, (**D**) AGG- and (**E**) GGG-enriched proteins. The D/U ratio of (**F**) CAA-, (**G**) GGA-, (**H**) AAA-, (**I**) AGG- and (**J**) GGG-enriched proteins.
(JPG)

S4 Fig Controlling for changes in mRNA expression, depletion of mcm^5^ and mcm^5^s^2^ still selectively repress expression of proteins enriched with AGA/GAA codons. Proteins whose changes in protein expression could possibly be explained by altered mRNA expression in *trm9*Δ cells were excluded from the analysis. (**A**) The percentage of down-regulated proteins of AGA-enriched proteins *vs* non-enriched proteins. The histogram shows the distribution of the percentages obtained by 100,000 random samplings of the non-enriched proteins. The position of AGA-enriched proteins to that distribution is indicated by arrow, with the p-values indicating the chance that a random sampling has a value no less than that of the AGA-enriched group. (**B**) Similar analysis performed for GAA-enriched proteins.
(JPG)

S5 Fig Controlling for protein expression, proteins enriched with AGA and GAA codons are still selectively down-regulated in *trm9*Δ cells. (**A**) A comparison of expression levels for AGA-enriched proteins *vs* non-enriched proteins. Protein abundance was estimated using summed peptide intensity from the mass spectrometric analysis. P-value was calculated using student’s t-test. (**B**) A comparison of expression levels for AGA-enriched proteins *vs* non-enriched proteins. P-value was calculated using student’s t-test. (**C**) D/U ratios of AGA-enriched proteins *vs* non-enriched proteins controlling for protein expression. Proteins were binned into 10 groups based on expression level, and random sampling of non-enriched population were performed keeping the composition of proteins with different expression levels identical to that of AGA-enriched group. The histogram shows the distribution of D/U ratios of the non-enriched proteins obtained by 100,000 random samplings, the positions of AGA-enriched proteins are indicated by arrow, with the p-values indicating the chance that a random sampling has a ratio no less than that of the AGA/GAA-enriched group. (**D**) D/U ratios of GAA-enriched proteins *vs* non-enriched proteins controlling for protein expression, as described above.
(JPG)

S6 Fig Proteins enriched with AGA and GAA codons are selectively down-regulated in *trm9*Δ cells in response to MMS treatment. (**A**) Proteins were divided into seven groups based on AGA codon frequency or (**B**) GAA codon frequency, and D/U ratios were calculated in each group, respectively. The plots show a positive correlation between down-regulation and AGA or GAA codon content. (**C**) D/U ratios of AGA-enriched proteins *vs* non-enriched proteins. The histogram shows the distribution of D/U ratios of the non-enriched proteins obtained by 100,000 random samplings, the positions of AGA-enriched proteins are indicated by arrow, with the p-values indicating the chance that a random sampling has a ratio no less than that of the AGA/GAA-enriched group. (**D**) D/U ratios of GAA-enriched proteins *vs* non-enriched proteins, as described above.
(JPG)

S7 Fig AGA/GAA codon runs occur more frequently than expected by chance. (**A**) Boxplots show the number of 4-mer codon runs identified in each gene (actual) *vs* that of 10,000 shufflsed sequences per gene maintaining the codon composition of each gene (randomized). P-value was calculated by Mann-Whitney U test. (**B**) Similar to (A), but 5-mer codon runs were considered.
(JPG)

S8 Fig Biological processes affected by Trm9-dependent proteins under normal growth conditions and following MMS treatment. Gene Ontology category enrichment was determined for down- or up-regulated in *trm9*Δ cells compared to wild-type cells under normal growth conditions and following MMS treatment.
(JPG)

S1 Table Proteins quantified in the SILAC proteomics analysis of wild-type and *trm9Δ* yeast. (XLSX)

S2 Table Significantly up- and down-regulated proteins in *trm9Δ* cells under normal growth conditions and following DNA damage stress with MMS. (XLSX)

S3 Table Codon usage data for individual genes in *S*. *cerevisiae*. (XLSX)

S4 Table Function categories of differentially expressed proteins enriched with Arg^AGA^ and/or Glu^GAA^ codons in *trm9Δ* cell in normal and MMS conditions (DOCX)

S5 Table Function categories of differentially expressed proteins in *trm9Δ* cell in normal and MMS conditions. (DOCX)

S6 Table Comparison of published studies of wobble U modifications. (XLSX)

S7 Table Proteomic analysis in *trm9Δ* cells of genes conferring resistance to MMS exposure. (XLSX)
