# Supplementary figures and images for: Trm9-Catalyzed tRNA Modifications Regulate Global Protein Expression by Codon-Biased Translation
Source: PLoS Genet. 2015 Dec 15;11(12):e1005706. doi: 10.1371/journal.pgen.1005706 (PMC4689569; doi:10.1371/journal.pgen.1005706)

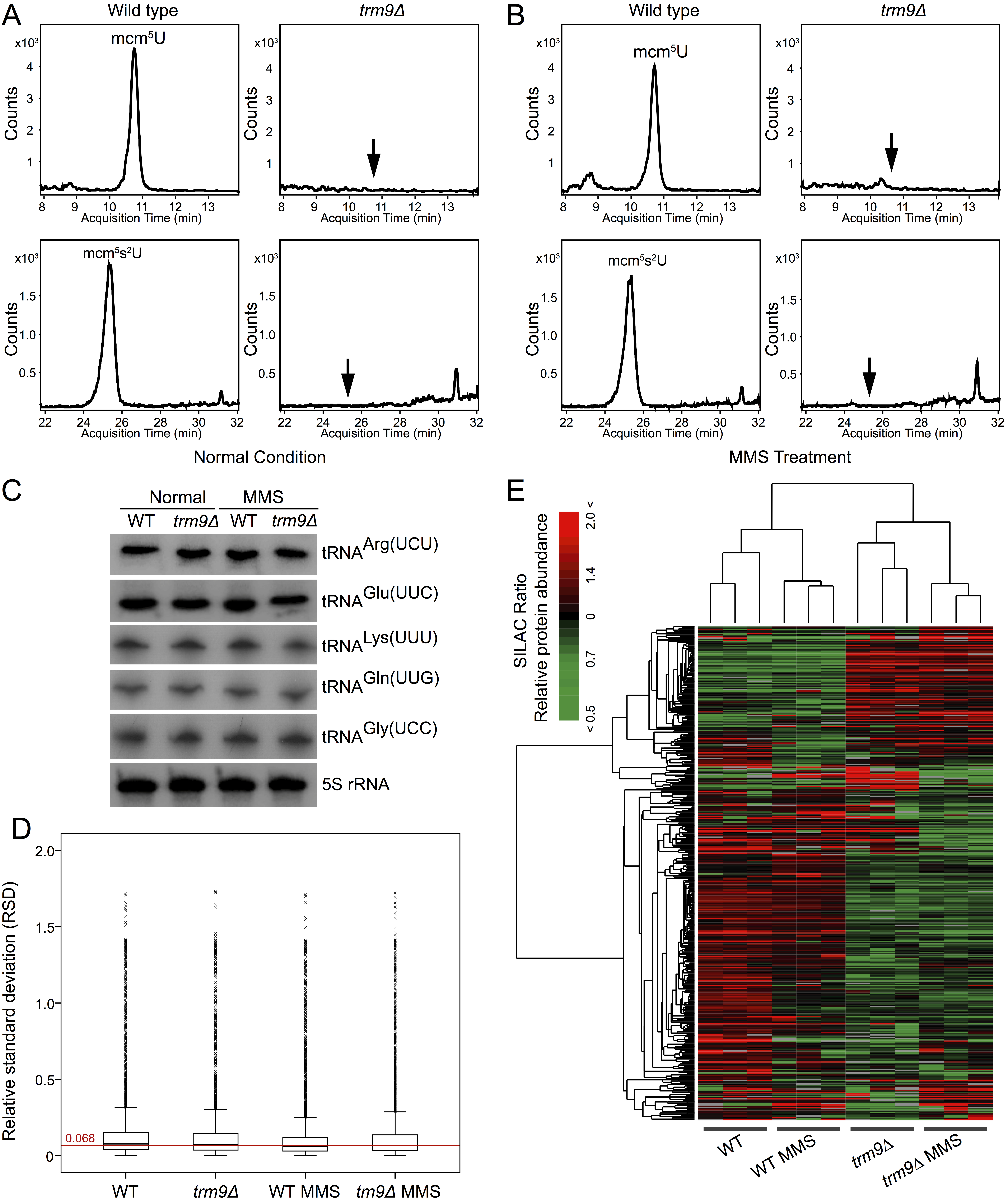

Supplement: S1 Fig — (A) The chromatograms indicate that depletion of Trm9 results in a total loss of the modified ribonucleosides mcm5U and mcm5s2U in yeast cells under normal conditions and (B) in response to MMS treatment. Modifications were identified by HPLC retention time and collision-induced dissociation fragmentation patterns. (C) Northern-blot analysis of tRNA abundance in wild-type (WT) and trm9Δ cells under normal condition and in response to MMS treatment. (D) Relative standard deviation (RSD) of SILAC ratios between three replicates of each sample. RSD is the absolute value of the coefficient of variation calculated by dividing the standard deviation by the mean value. The median RSD for SILAC quantification was 0.068. (E) Hierarchical clustering of SILAC ratios (which indicate relative protein abundance) of all proteins identified in replicates of WT and trm9Δ cells under normal and stress conditions. (JPG) [file pgen.1005706.s001.jpg]

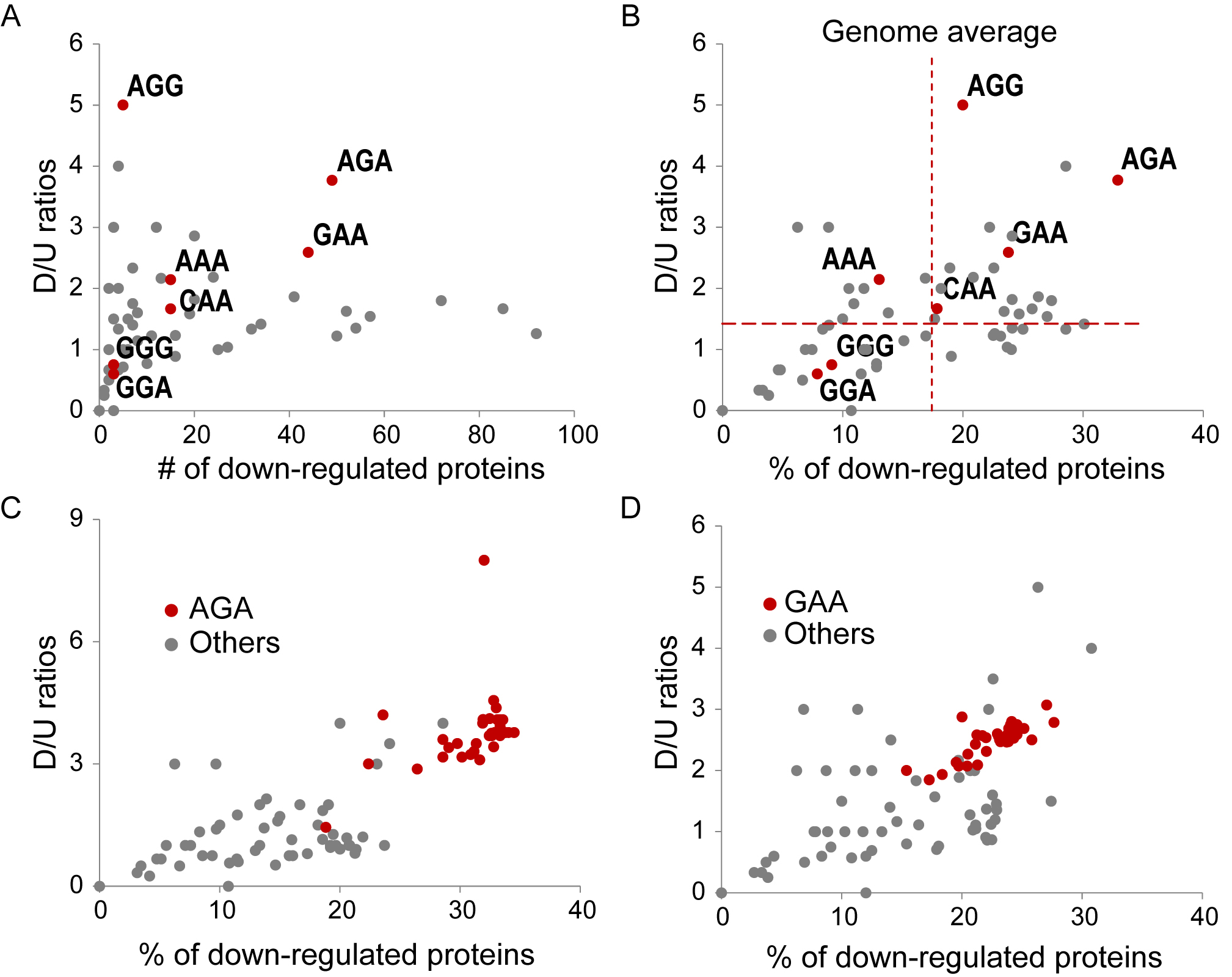

Supplement: S2 Fig — (A) Plot of D/U ratio versus the number of significantly down-regulated proteins in each group of proteins enriched with one certain codon is shown. The red dots represent codons dependent on the wobble modifications, while grey dots represent data of other codon groups. (B) Plot of D/U ratio versus the percentage of significantly down-regulated proteins in each group. The red line indicates the percentage of significantly down-regulated proteins in total proteins. (C) To control for biased usage of codons other than AGA (except for GAA), proteins enriched with other codons were removed from the AGA-enriched group, one at a time. Proteins enriched with AGA were likewise removed from other codon groups. The values of the proteins enriched with AGA to the exclusion of other codons were separately plotted in red dots, while the values of the proteins enriched with other codons, after removing AGA-enriched proteins, were plotted in grey dots. (D) Analysis similar to (C) repeated for proteins enriched with GAA codon. (JPG) [file pgen.1005706.s002.jpg]

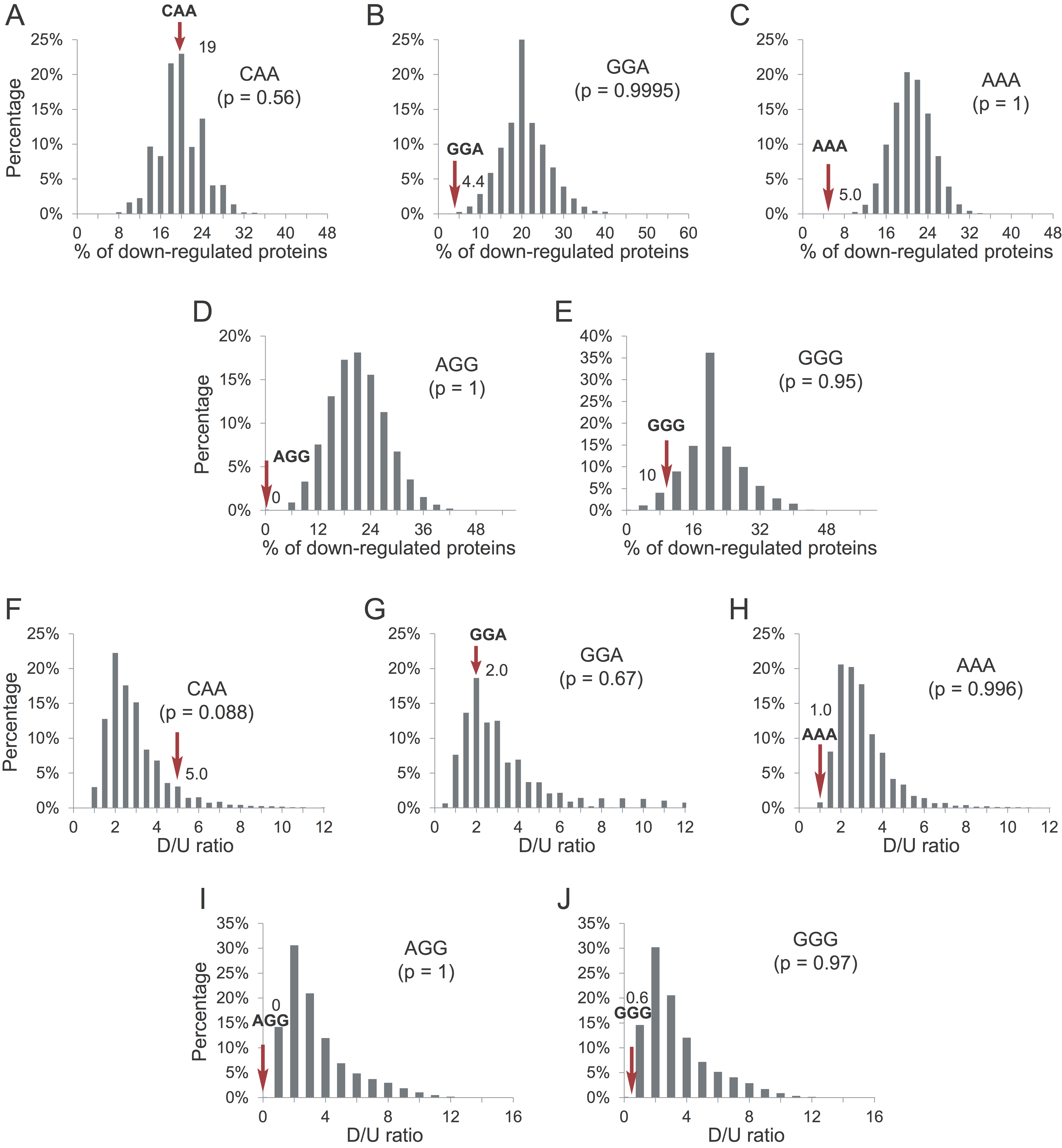

Supplement: S3 Fig — (A) The percentage of down-regulated proteins of CAA-enriched proteins vs non-enriched proteins. The histogram shows the distribution of the percentages obtained by 100,000 random samplings of the non-enriched proteins. The position of CAA-enriched proteins to that distribution is indicated by arrow, with the p-values indicating the chance that a random sampling has a value no less than that of the CAA-enriched group. Similar results were observed for (B) GGA-, (C) AAA-, (D) AGG- and (E) GGG-enriched proteins. The D/U ratio of (F) CAA-, (G) GGA-, (H) AAA-, (I) AGG- and (J) GGG-enriched proteins. (JPG) [file pgen.1005706.s003.jpg]

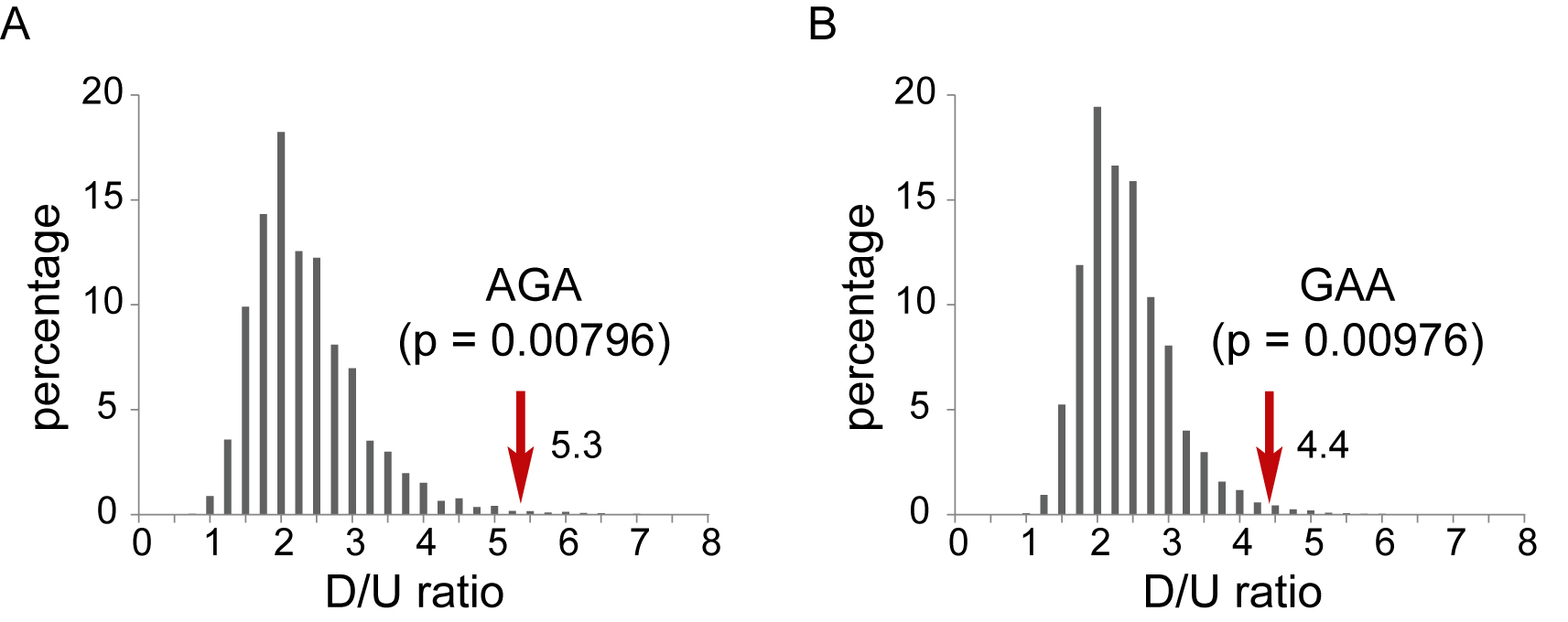

Supplement: S4 Fig — Proteins whose changes in protein expression could possibly be explained by altered mRNA expression in trm9Δ cells were excluded from the analysis. (A) The percentage of down-regulated proteins of AGA-enriched proteins vs non-enriched proteins. The histogram shows the distribution of the percentages obtained by 100,000 random samplings of the non-enriched proteins. The position of AGA-enriched proteins to that distribution is indicated by arrow, with the p-values indicating the chance that a random sampling has a value no less than that of the AGA-enriched group. (B) Similar analysis performed for GAA-enriched proteins. (JPG) [file pgen.1005706.s004.jpg]

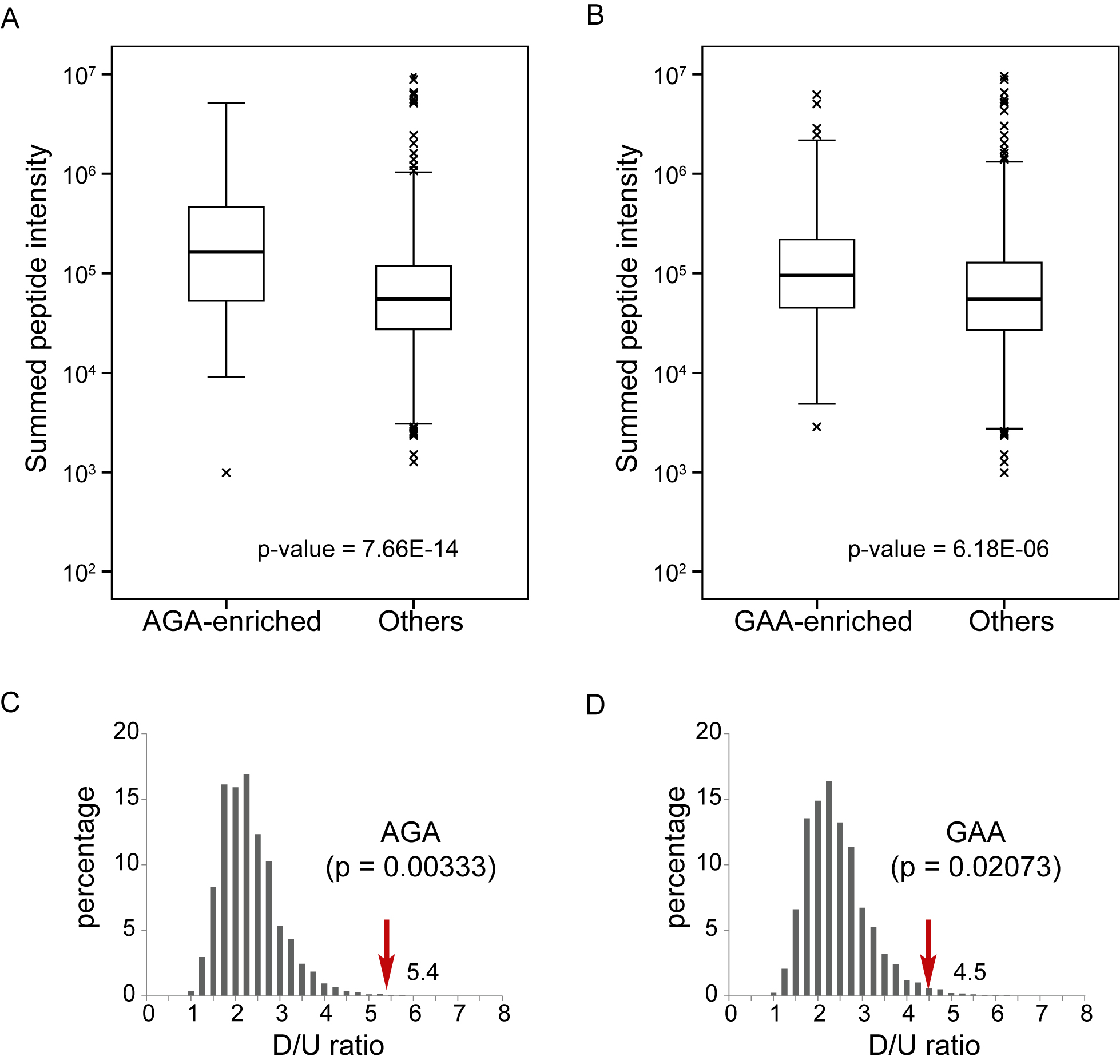

Supplement: S5 Fig — (A) A comparison of expression levels for AGA-enriched proteins vs non-enriched proteins. Protein abundance was estimated using summed peptide intensity from the mass spectrometric analysis. P-value was calculated using student’s t-test. (B) A comparison of expression levels for AGA-enriched proteins vs non-enriched proteins. P-value was calculated using student’s t-test. (C) D/U ratios of AGA-enriched proteins vs non-enriched proteins controlling for protein expression. Proteins were binned into 10 groups based on expression level, and random sampling of non-enriched population were performed keeping the composition of proteins with different expression levels identical to that of AGA-enriched group. The histogram shows the distribution of D/U ratios of the non-enriched proteins obtained by 100,000 random samplings, the positions of AGA-enriched proteins are indicated by arrow, with the p-values indicating the chance that a random sampling has a ratio no less than that of the AGA/GAA-enriched group. (D) D/U ratios of GAA-enriched proteins vs non-enriched proteins controlling for protein expression, as described above. (JPG) [file pgen.1005706.s005.jpg]

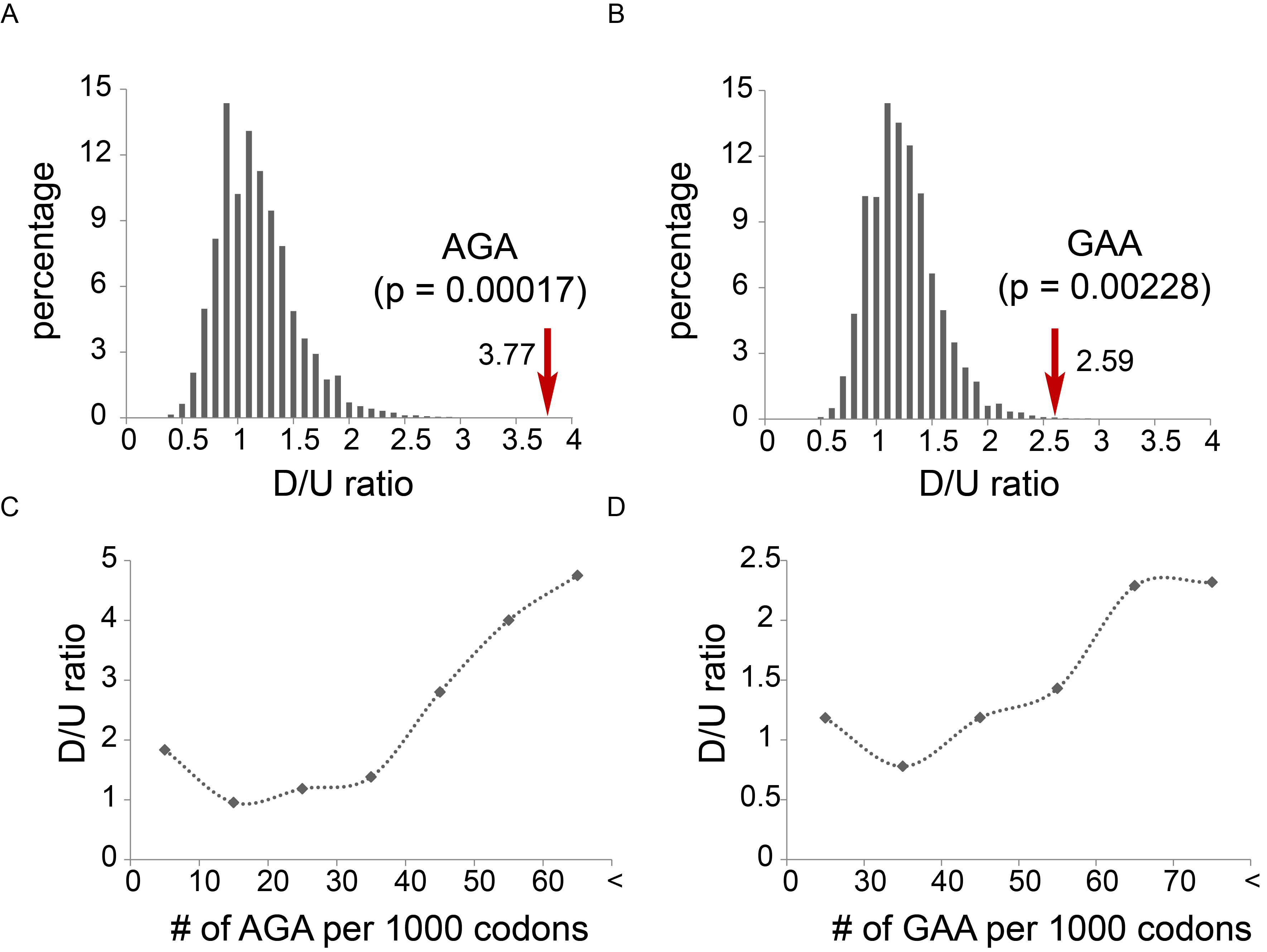

Supplement: S6 Fig — (A) Proteins were divided into seven groups based on AGA codon frequency or (B) GAA codon frequency, and D/U ratios were calculated in each group, respectively. The plots show a positive correlation between down-regulation and AGA or GAA codon content. (C) D/U ratios of AGA-enriched proteins vs non-enriched proteins. The histogram shows the distribution of D/U ratios of the non-enriched proteins obtained by 100,000 random samplings, the positions of AGA-enriched proteins are indicated by arrow, with the p-values indicating the chance that a random sampling has a ratio no less than that of the AGA/GAA-enriched group. (D) D/U ratios of GAA-enriched proteins vs non-enriched proteins, as described above. (JPG) [file pgen.1005706.s006.jpg]

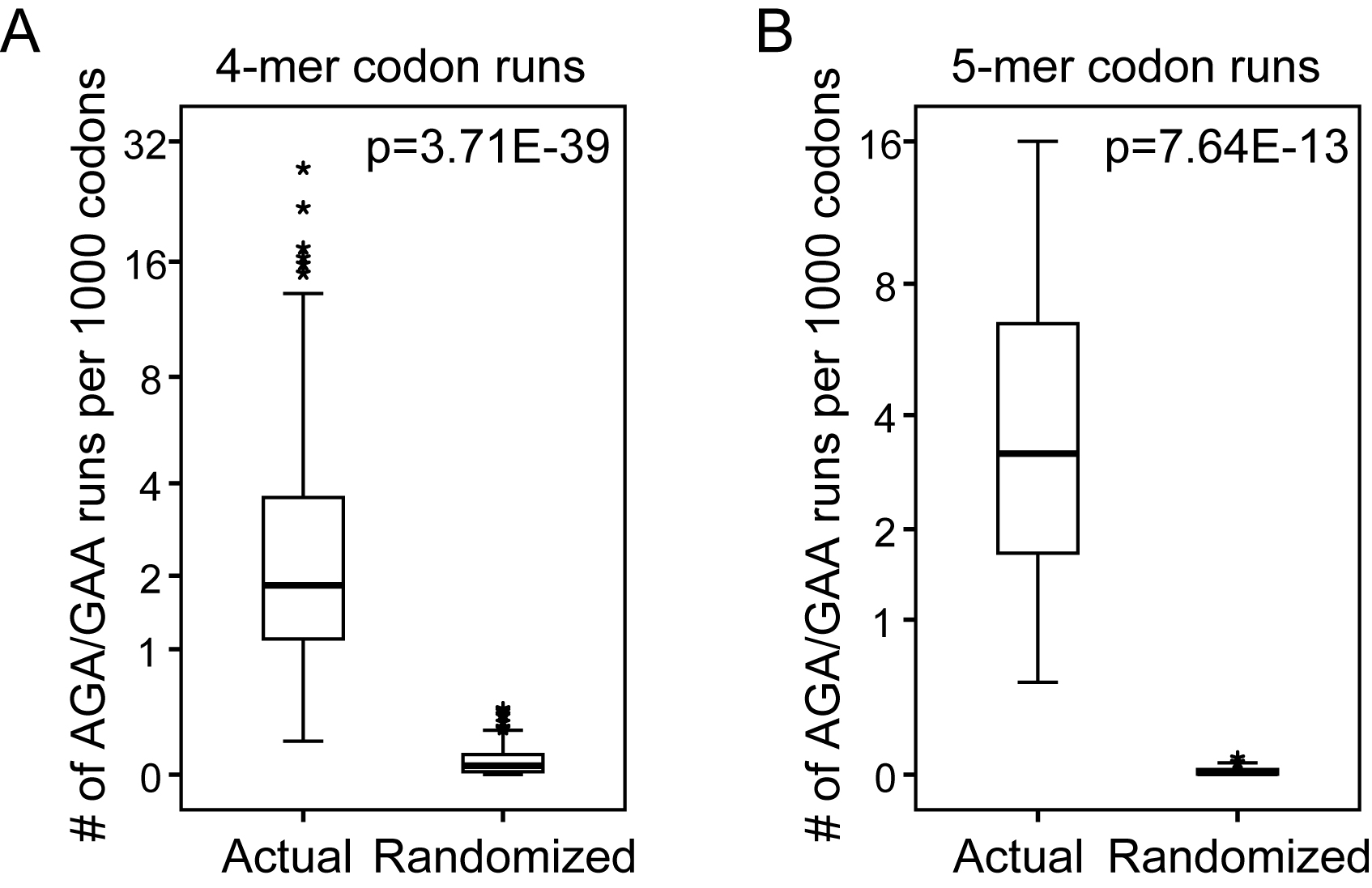

Supplement: S7 Fig — (A) Boxplots show the number of 4-mer codon runs identified in each gene (actual) vs that of 10,000 shufflsed sequences per gene maintaining the codon composition of each gene (randomized). P-value was calculated by Mann-Whitney U test. (B) Similar to (A), but 5-mer codon runs were considered. (JPG) [file pgen.1005706.s007.jpg]

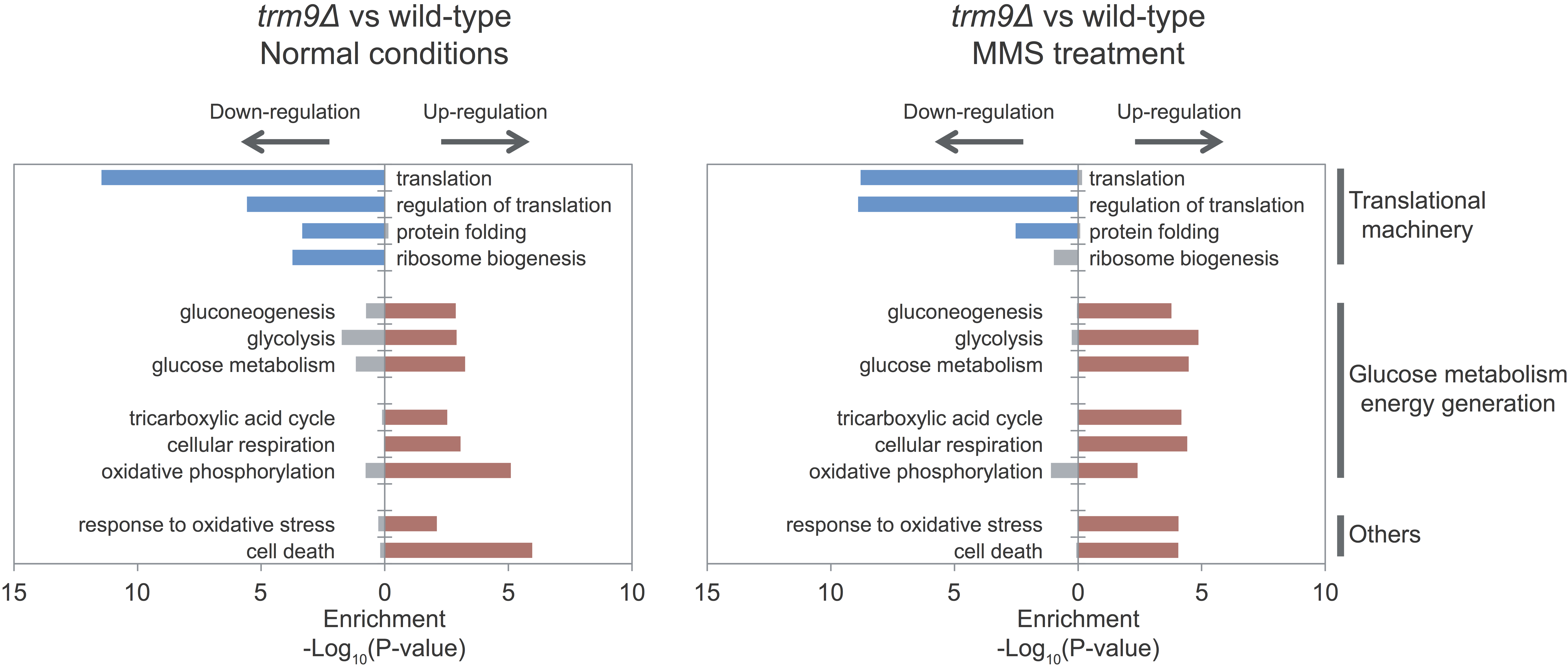

Supplement: S8 Fig — Gene Ontology category enrichment was determined for down- or up-regulated in trm9Δ cells compared to wild-type cells under normal growth conditions and following MMS treatment. (JPG) [file pgen.1005706.s008.jpg]
