# Supplementary material for: Trm9-Catalyzed tRNA Modifications Regulate Global Protein Expression by Codon-Biased Translation
Source: PLoS Genet. 2015 Dec 15;11(12):e1005706. doi: 10.1371/journal.pgen.1005706 (PMC4689569; doi:10.1371/journal.pgen.1005706)
Supplement: S4 Table — (DOCX) [file pgen.1005706.s012.docx]

**Table S4**. Function categories of differentially expressed proteins enriched with Arg^AGA^ and/or Glu^GAA^ codons in *trm9Δ* cell in normal and MMS conditions

| **GO-ID**  **Description** | **Normal** | | **MMS Treatment** | |
| --- | --- | --- | --- | --- |
|  | **Up-Regulated Proteins** | **Down-Regulated Proteins** | **Up-Regulated Proteins** | **Down-Regulated Proteins** |
|  | **Percentage (Enrichment)**  **Proteins** | **Percentage (Enrichment)**  **Proteins** | **Percentage (Enrichment)**  **Proteins** | **Percentage (Enrichment)**  **Proteins** |
| GO: 0006412  translation | 17.65% (5.01e-01) | 64.20% (1.76e-23) | 17.86% (4.40e-01) | 67.11% (2.06e-24) |
|  | Leu1, Caf20, Tif3 | Krs1, Rpl36a, Rps2, Rps3, Rpl9b, Sui2, Rpl9a, Rpp2b, Grs1, Rpl10, Rpl19b, Rps23a, Efb1, Rpl35b, Rps9b, Rnr4, Rps0b, Rps0a, Eft2, Rpl31a, Rps16b, Rpl16a, Rpl16b, Rpl11b, Srp1, Hsc82, Rps15, Rps12, Tif2, Rps13, Rpl20a, Acb1, Rps1a, Rps1b, Tef2, Rps20, Rpl7a, Ilv6, Met6, Hsp104, Rps19b, Rps10b, Rpp0, Rpl33b, Ssa1, Ssa2, Rps7a, Rpl40a, Rps6b, Gln1, Rps31, Rps18a | Leu1, Rpl35b, Tuf1, Vas1, Rpl26b | Krs1, Rps8a, Rps17b, Yef3, Apa1, Sui2, Rpl9a, Rpp2b, Rpp2a, Rpl10, Rpl19b, Ssb1, Rps7b, Efb1, Rps9b, Rpl4a, Rnr4, Eft2, Rpl31a, Dps1, Rps16b, Rpl16b, Hsc82, Rpp1a, Rpp1b, Rps4b, Rps12, Tif2, Rpl8b, Rps22a, Acb1, Rpl38, Rps1a, Rps1b, Rpl17b, Rpl14b, Rps29a, Rps19b, Rps10b, Ssa1, Rps5, Ssa2, Rpl40a, Rpl33a, Stm1, Ded1, Tif4631, Rps6b, Rpl18b, Rpl13b, Gln1 |
| **GO-ID**  **Description** | **Normal** | | **MMS Treatment** | |
|  | **Up-Regulated Proteins** | **Down-Regulated Proteins** | **Up-Regulated Proteins** | **Down-Regulated Proteins** |
|  | **Percentage (Enrichment)**  **Proteins** | **Percentage (Enrichment)**  **Proteins** | **Percentage (Enrichment)**  **Proteins** | **Percentage (Enrichment)**  **Proteins** |
| GO: 0006417  regulation of translation | 17.65% (1.44e-02) | 28.40% (1.02e-16) | 10.71% (5.42e-02) | 38.16% (4.70e-25) |
|  | Caf20, Fba1, Sec53 | Rps9b, Rpl20a, Cdc19, Pfk2, Rps0b, Ola1, Eft2, Rps0a, Rps1a, Rps2, Rpl31a, Aro4, Rps1b, Rps16b, Sui2, Rpl16a, Zuo1, Tef2, Grs1, Tif2, Rpl10, Rpl7a, Rps23a | Fba1, Vas1, Sec53 | Rps8a, Sbp1, Yef3, Cdc19, Pfk1, Rps1a, Rps1b, Sui2, Rpl17b, Rpl14b, Rpl10, Ssb1, Rps9b, Rpl4a, Eft2, Rpl31a, Kap123, Rps5, Dps1, Rps16b, Stm1, Tif4631, Zuo1, Rpl18b, Rpl13b, Rps4b, Tif2, Gcn20, Rpl8b |
| GO: 0042254  ribosome biogenesis | 5.88% (6.51e-01) | 33.33% (4.61e-14) | 7.14% (5.07e-01) | 25.00% (6.15e-08) |
|  | Lsg1 | Rps2, Rps1a, Rps3, Rps1b, Rpl9b, Drs1, Rpl10, Rps23a, Rps20, Rps19b, Rpl35b, Rps9b, Rps10b, Rps0b, Rps0a, Rpp0, Rpl31a, Rps7a, Rpl40a, Rps16b, Rps6b, Rpl11b, Rps15, Rps31, Rps13, Nop58, Rps18a | Rpl35b, Gar1 | Rps8a, Rps17b, Rps9b, Rps10b, Rpl38, Rpl31a, Rps1a, Rps5, Rpl40a, Rps16b, Yrb1, Rps1b, Rps6b, Tif4631, Drs1, Rpl10, Rpl8b, Rps19b, Rps7b |
| **GO-ID**  **Description** | **Normal** | | **MMS Treatment** | |
|  | **Up-Regulated Proteins** | **Down-Regulated Proteins** | **Up-Regulated Proteins** | **Down-Regulated Proteins** |
|  | **Percentage (Enrichment)**  **Proteins** | **Percentage (Enrichment)**  **Proteins** | **Percentage (Enrichment)**  **Proteins** | **Percentage (Enrichment)**  **Proteins** |
| GO: 0006364  rRNA processing | 0.00% (1.00e+00) | 20.99% (1.61e-08) | 7.14% (3.15e-01) | 10.53% (1.15e-02) |
|  |  | Rps9b, Rpl35b, Rps0b, Rps0a, Rps2, Rps1a, Rps7a, Rps16b, Rps1b, Rps6b, Drs1, Rps31, Nop58, Rps13, Rps23a, Rps20, Rps18a | Rpl35b, Gar1 | Rps8a, Rps6b, Drs1, Rps9b, Rps1a, Rps1b, Rps16b, Rps7b |
| GO: 0034660  ncRNA metabolic process | 0.00% (1.00e+00) | 23.46% (8.03e-07) | 10.71% (2.80e-01) | 13.16% (2.65e-02) |
|  |  | Krs1, Rps9b, Rpl35b, Rps0b, Rps0a, Rps2, Rps1a, Rps7a, Rps16b, Rps1b, Rps6b, Drs1, Grs1, Rps31, Nop58, Rps13, Rps20, Rps23a, Rps18a | Rpl35b, Gar1, Vas1 | Rps8a, Krs1, Rps6b, Drs1, Rps9b, Rps1a, Dps1, Rps1b, Rps16b, Rps7b |
| GO: 0006457  protein folding | 0.00% (1.00e+00) | 12.35% (1.70e-06) | 3.57% (4.03e-01) | 10.53% (6.34e-05) |
|  |  | Zuo1, Ydj1, Hsc82, Sse1, Hsp60, Sti1, Ssa1, Ssa2, Fpr4, Hsp104 | Kar2 | Zuo1, Hsc82, Sse1, Ssa1, Ssa2, Hsp82, Fpr4, Ssb1 |
| **GO-ID**  **Description** | **Normal** | | **MMS Treatment** | |
|  | **Up-Regulated Proteins** | **Down-Regulated Proteins** | **Up-Regulated Proteins** | **Down-Regulated Proteins** |
|  | **Percentage (Enrichment)**  **Proteins** | **Percentage (Enrichment)**  **Proteins** | **Percentage (Enrichment)**  **Proteins** | **Percentage (Enrichment)**  **Proteins** |
| GO:0006974  response to DNA damage stimulus | 11.76% (2.08e-01) | 1.23% (9.85e-01) | 3.57% (7.64e-01) | 3.95% (7.42e-01) |
|  | Hta2, Smc1 | Rpl40a | Tuf1 | Def1, Mph1, Rpl40a |
| GO:0007049  cell cycle | 5.88% (8.27e-01) | 2.47% (9.98e-01) | 0.00% (1.00e+00) | 5.26% (9.48e-01) |
|  | Smc1 | Cmd1, Myo2 |  | Nbp1, Cmd1, Yrb1, Myo1 |
| GO: 0019319  hexose biosynthetic process | 17.65% (1.89e-05) | 1.23% (2.31e-01) | 10.71% (8.90e-05) | 0.00% (1.00e+00) |
|  | Gpm1, Fba1, Sec53 | Pyc2 | Gpm1, Fba1, Sec53 |  |
| GO: 0006091  generation of precursor metabolites and energy | 29.41% (4.98e-04) | 3.70% (6.64e-01) | 17.86% (5.43e-03) | 3.95% (6.21e-01) |
|  | Atp1, Atp2, Gsy2, Gpm1, Fba1 | Vma2, Cdc19, Pfk2 | Atp1, Atp2, Gsy2, Gpm1, Fba1 | Vma2, Cdc19, Pfk1 |
| GO: 0006094  gluconeogenesis | 11.76% (9.37e-04) | 1.23% (2.00e-01) | 7.14% (2.56e-03) | 0.00% (1.00e+00) |
|  | Gpm1, Fba1 | Pyc2 | Gpm1, Fba1 |  |
| GO: 0009408  response to heat | 11.76% (2.04e-03) | 2.47% (4.16e-02) | 10.71% (1.77e-04) | 0.00% (1.00e+00) |
|  | Lsp1, Hsp12 | Ydj1, Hsp104 | Lsp1, Pil1, Hsp12 |  |
| **GO-ID**  **Description** | **Normal** | | **MMS Treatment** | |
|  | **Up-Regulated Proteins** | **Down-Regulated Proteins** | **Up-Regulated Proteins** | **Down-Regulated Proteins** |
|  | **Percentage (Enrichment)**  **Proteins** | **Percentage (Enrichment)**  **Proteins** | **Percentage (Enrichment)**  **Proteins** | **Percentage (Enrichment)**  **Proteins** |
| GO: 0006096  glycolysis | 11.76% (3.13e-03) | 2.47% (6.13e-02) | 7.14% (8.42e-03) | 2.63% (5.48e-02) |
|  | Gpm1, Fba1 | Cdc19, Pfk2 | Gpm1, Fba1 | Cdc19, Pfk1 |
| GO: 0016265  death | 11.76% (8.64e-03) | 0.00% (1.00e+00) | 7.14% (2.26e-02) | 0.00% (1.00e+00) |
|  | Atp1, Atp2 |  | Atp1, Atp2 |  |

“Percentage”: the percentage of significantly changed proteins involved in each GO category.

“Enrichment”: the enrichment of significantly changed proteins in each GO category (hypergeometric distribution).
