# Supplementary material for: Trm9-Catalyzed tRNA Modifications Regulate Global Protein Expression by Codon-Biased Translation
Source: PLoS Genet. 2015 Dec 15;11(12):e1005706. doi: 10.1371/journal.pgen.1005706 (PMC4689569; doi:10.1371/journal.pgen.1005706)
Supplement: S5 Table — (DOCX) [file pgen.1005706.s013.docx]

**Table S5**. Function categories of differentially expressed proteins in *trm9Δ* cell in normal and MMS conditions

| **GO-ID**  **Description** | **Normal** | | **MMS Treatment** | |
| --- | --- | --- | --- | --- |
|  | **Up-Regulated Proteins** | **Down-Regulated Proteins** | **Up-Regulated Proteins** | **Down-Regulated Proteins** |
|  | **Percentage (Enrichment)**  **Proteins** | **Percentage (Enrichment)**  **Proteins** | **Percentage (Enrichment)**  **Proteins** | **Percentage (Enrichment)**  **Proteins** |
| GO:0006412  translation | 10.53% (9.97e-01) | 37.39% (7.81e-12) | 18.25% (7.73e-01) | 36.41% (4.98e-09) |
|  | Arg1, Leu1, Nab6, Lys20, Caf20, Gly1, Tif3, Cwp1, Mmf1, Tma19 | Krs1, Rpl36a, Sis1, Oye2, Ded81, Rpl1b, Age2, Rps2, Rps3, Sui1, Rpl9b, Rpl12b, Rli1, Sui2, Rpl9a, Rpp2b, Grs1, Rpl10, Rps23a, Rpl19b, Cys3, Efb1, Rpl35b, Rps9b, Dld3, Rnr4, Ngl1, Rps0b, Rps0a, Eft2, Rpl31a, Tys1, Rps16b, Rpl16a, Rpl16b, Rpl11b, Hsc82, Srp1, Rps15, Rps12, Tif2, Rps13, Yel047c, Rps21b, Rpl20a, Ydr341c, Acb1, Idp1, Adh5, Rpl27a, Cam1, Hts1, Wrs1, Rps1a, Dhh1, Rps1b, Rpl30, Arg4, Tef4, Tef2, Smt3, Rpl7a, Rps20, Met6, Ilv6, Rps19b, Hsp104, Hom3, Rps10b, His5, Ils1, Rpl33b, Rpp0, Ynl247w, Ssa1, Ssa2, Rps7a, Rpl40a, Rps6b, Gln1, Rpl6a, Rps28b, Dpm1, Rps31, Rps18a, Met14 | Leu1, Pir1, Cdc13, Vas1, Tma19, Rpl26b, Arg1, Tdh3, Aac3, Adh2, Mrpl19, Mmf1, Rpl35b, Bgl2, Mir1, Cwp1, Rpl25, Hmo1, Lys20, Hyp2, Tuf1, Gnd1, Dbp5, Hef3, Met13 | Krs1, Rps8a, Rps17b, Yef3, Oye2, Apa1, Ded81, Rpl1b, Sui1, Sui2, Rpl9a, Rpp2b, Rpp2a, Hnt1, Rpl10, Rpl19b, Ssb1, Cys3, Rps7b, Efb1, Rps9b, Dld3, Rpl4a, Rnr4, Eft2, Rpl31a, Tys1, Dps1, Rps16b, Rpl16b, Hsc82, Rpp1a, Rps4b, Rpp1b, Rps12, Tif2, Ggc1, Rps21b, Rpl8b, Rps22a, Sst2, Ydr341c, Acb1, Cam1, Hts1, Wrs1, Rpl38, Rps1a, Rps1b, Sro9, Hor2, Rpl17b, Tef4, Rpl14b, Rps29a, Rps19b, Rps10b, Ils1, Ssa1, Ssa2, Rps5, Rpl33a, Rpl40a, Stm1, Ded1, Rps6b, Tif4631, Rpl18b, Rpl13b, Gus1, Gln1 |
| **GO-ID**  **Description** | **Normal** | | **MMS Treatment** | |
|  | **Up-Regulated Proteins** | **Down-Regulated Proteins** | **Up-Regulated Proteins** | **Down-Regulated Proteins** |
|  | **Percentage (Enrichment)**  **Proteins** | **Percentage (Enrichment)**  **Proteins** | **Percentage (Enrichment)**  **Proteins** | **Percentage (Enrichment)**  **Proteins** |
| GO:0006417  regulation of translation | 4.21% (9.87e-01) | 17.83% (8.19e-06) | 5.84% (9.77e-01) | 22.56% (5.10e-09) |
|  | Caf20, Psa1, Fba1, Sec53 | Rpl20a, Cdc19, Pfk2, Wrs1, Pub1, Rps2, Rps1a, Dhh1, Aro4, Rps1b, Sui1, Rpl12b, Sui2, Rli1, Rpl30, Tef4, Tef2, Grs1, Rpl10, Rps23a, Rpl7a, Ade3, Snf1, Rps9b, His4, Ils1, Rps0b, Asc1, Rps0a, Ola1, Eft2, Imd3, Imd2, Rpl31a, Rps16b, Fas1, Rpl16a, Zuo1, Thr4, Rpl6a, Tif2 | Psa1, Hyp2, Fba1, Vas1, Pdc1, Ura2, Sec53, Yih1 | Rps8a, Sbp1, Tpd3, Yef3, Cdc19, Pfk1, Wrs1, Pub1, Rps1a, Rps1b, Sro9, Sui1, Sui2, Rpl17b, Tef4, Rpl14b, Rpl10, Ade3, Ssb1, Rps9b, Rpl4a, His4, Nat1, Ils1, Asc1, Eft2, Imd3, Imd2, Rpl31a, Kap123, Rps5, Dps1, Stm1, Rps16b, Tif4631, Zuo1, Rpl18b, Thr4, Rpl13b, Gus1, Rps4b, Tif2, Rpl8b, Gcn20 |
| GO:0042254  ribosome biogenesis | 4.21% (9.89e-01) | 16.09% (5.52e-04) | 3.65% (1.00e+00) | 12.82% (1.58e-01) |
|  | Rrp40, Urb1, Lsg1, Dbp9 | Gsp1, Cam1, Rps1a, Rps2, Rps3, Rps1b, Rpl9b, Rpl12b, Rli1, Rpl30, Drs1, Rpl10, Rps23a, Rps20, Rps19b, Rps9b, Rpl35b, Rps10b, Rps0b, Rpp0, Rps0a, Rpl31a, Pih1, Rps7a, Rpl40a, Rps16b, Rps6b, Rpl11b, Rps15, Rps28b, Rpl6a, Rps31, Rps13, Nop58, Prp43, Rps18a, Rps21b | Rpl35b, Drs2, Ytm1, Gar1, Rpl25 | Rps8a, Gsp1, Rps17b, Dia2, Afg2, Cam1, Rpl38, Rps1a, Rps1b, Yrb1, Drs1, Rpl10, Rps19b, Rps7b, Rps9b, Rps10b, Rpl31a, Rps5, Rpl40a, Rps16b, Tif4631, Rps6b, Rps21b, Rpl8b, Dbp9 |
| **GO-ID**  **Description** | **Normal** | | **MMS Treatment** | |
|  | **Up-Regulated Proteins** | **Down-Regulated Proteins** | **Up-Regulated Proteins** | **Down-Regulated Proteins** |
|  | **Percentage (Enrichment)**  **Proteins** | **Percentage (Enrichment)**  **Proteins** | **Percentage (Enrichment)**  **Proteins** | **Percentage (Enrichment)**  **Proteins** |
| GO:0034660  ncRNA metabolic process | 3.16% (9.91e-01) | 13.91% (1.15e-03) | 2.92% (1.00e+00) | 10.77% (3.76e-01) |
|  | Rrp40, Urb1, Dbp9 | Krs1, Gsp1, Ydr341c, Ded81, Hts1, Wrs1, Rps1a, Rps2, Rps1b, Rli1, Rpl30, Drs1, Grs1, Rps23a, Rps20, Rps9b, Rpl35b, Ils1, Rps0b, Rps0a, Ynl247w, Pih1, Tys1, Rps7a, Rps16b, Rps6b, Rps31, Rps13, Nop58, Prp43, Rps18a, Rps21b | Rpl35b, Ytm1, Gar1, Vas1 | Krs1, Rps8a, Gsp1, Rps9b, Ydr341c, Ils1, Ded81, Hts1, Wrs1, Rps1a, Tys1, Dps1, Rps16b, Rps1b, Rps6b, Drs1, Deg1, Gus1, Rps21b, Rps7b, Dbp9 |
| GO:0006457  protein folding | 0.00% (1.00e+00) | 7.83% (1.67e-03) | 2.19% (9.37e-01) | 6.67% (4.38e-02) |
|  |  | Sis1, Ydj1, Egd1, Hsp60, Sti1, Cct3, Ssa1, Hsp10, Pih1, Ssa2, Fpr4, Cct6, Zuo1, Cpr5, Cpr6, Hsc82, Sse1, Hsp104 | Hsp26, Kar2, Cpr1 | Ssa1, Hsp10, Ssa2, Fpr4, Hsp82, Ssa4, Zuo1, Cpr6, Hsc82, Sse1, Egd2, Emc1, Ssb1 |
| **GO-ID**  **Description** | **Normal** | | **MMS Treatment** | |
|  | **Up-Regulated Proteins** | **Down-Regulated Proteins** | **Up-Regulated Proteins** | **Down-Regulated Proteins** |
|  | **Percentage (Enrichment)**  **Proteins** | **Percentage (Enrichment)**  **Proteins** | **Percentage (Enrichment)**  **Proteins** | **Percentage (Enrichment)**  **Proteins** |
| GO:0006091  generation of precursor metabolites and energy | 26.32% (1.20e-08) | 9.57% (1.99e-01) | 22.63% (6.95e-09) | 5.64% (9.40e-01) |
|  | Atp19, Gsy2, Pet9, Glk1, Tdh1, Atp3, Cit1, Atp4, Atp1, Nde1, Atp2, Adh1, Qcr2, Fba1, Idh2, Idh1, Cor1, Atp5, Eno1, Gpm1, Gph1, Pgm2, Pho85, Tps1, Mdh1 | Cdc19, Trx1, Idp1, Pfk2, Adh5, Hxk2, Pda1, Pdc5, Pdb1, Err3, Pah1, Vma2, Vma5, Tdh2, Vma7, Fre2, Eno2, Gdb1, Ugp1, Vma10, Snf1, Vma1 | Atp19, Gsy2, Pet9, Trx2, Glk1, Tdh1, Cit1, Tdh3, Atp1, Aac3, Atp2, Adh2, Adh1, Lsc1, Lsc2, Qcr2, Fba1, Idh2, Atp5, Eno1, Dld1, Aco1, Gpm1, Kgd1, Pdc1, Hxk1, Pgm2, Pgm1, Pgk1, Tps1, Mdh1 | Vph1, Vma2, Vma5, Hor2, Rhr2, Trx1, Cdc19, Pfk1, Hxk2, Vma10, Vma13 |
| GO:0009063  cellular amino acid catabolic process | 7.37% (1.26e-03) | 1.74% (6.61e-01) | 5.11% (7.00e-03) | 0.51% (9.79e-01) |
|  | Adh1, Put2, Gly1, Gcv1, Gad1, Car2, Bat1 | Lap3, Adh5, Pdc5, Ilv1 | Adh2, Gcv2, Adh1, Pdc1, Gcv1, Gad1, Car2 | Gdh2 |
| GO:0045333  cellular respiration | 7.37% (4.37e-03) | 0.87% (9.80e-01) | 8.03% (2.17e-04) | 0.00% (1.00e+00) |
|  | Cit1, Idh2, Qcr2, Pet9, Idh1, Cor1, Mdh1 | Pah1, Idp1 | Cit1, Dld1, Aac3, Aco1, Lsc1, Lsc2, Kgd1, Idh2, Qcr2, Pet9, Mdh1 |  |
| **GO-ID**  **Description** | **Normal** | | **MMS Treatment** | |
|  | **Up-Regulated Proteins** | **Down-Regulated Proteins** | **Up-Regulated Proteins** | **Down-Regulated Proteins** |
|  | **Percentage (Enrichment)**  **Proteins** | **Percentage (Enrichment)**  **Proteins** | **Percentage (Enrichment)**  **Proteins** | **Percentage (Enrichment)**  **Proteins** |
| GO:0006096  glycolysis | 6.32% (6.67e-03) | 3.48% (5.30e-02) | 7.30% (9.90e-05) | 1.54% (7.68e-01) |
|  | Tdh1, Gpm1, Fba1, Glk1, Eno1, Mdh1 | Tdh2, Cdc19, Eno2, Pfk2, Hxk2, Pda1, Pdb1, Err3 | Tdh1, Tdh3, Gpm1, Kgd1, Fba1, Hxk1, Pgk1, Glk1, Eno1, Mdh1 | Cdc19, Pfk1, Hxk2 |
| GO:0006094  gluconeogenesis | 4.21% (1.07e-02) | 1.30% (4.05e-01) | 4.38% (1.51e-03) | 0.00% (1.00e+00) |
|  | Tdh1, Gpm1, Fba1, Eno1 | Tdh2, Eno2, Pyc2 | Tdh1, Tdh3, Gpm1, Fba1, Pgk1, Eno1 |  |
| GO:0009109  coenzyme catabolic process | 4.21% (2.46e-02) | 0.87% (8.09e-01) | 5.11% (9.90e-04) | 0.00% (1.00e+00) |
|  | Cit1, Idh2, Idh1, Mdh1 | Idp1, Dug1 | Cit1, Aco1, Lsc1, Lsc2, Kgd1, Idh2, Mdh1 |  |
| GO:0006099  tricarboxylic acid cycle | 4.21% (1.92e-02) | 0.43% (9.48e-01) | 5.11% (5.88e-04) | 0.00% (1.00e+00) |
|  | Cit1, Idh2, Idh1, Mdh1 | Idp1 | Cit1, Aco1, Lsc1, Lsc2, Kgd1, Idh2, Mdh1 |  |
| **GO-ID**  **Description** | **Normal** | | **MMS Treatment** | |
|  | **Up-Regulated Proteins** | **Down-Regulated Proteins** | **Up-Regulated Proteins** | **Down-Regulated Proteins** |
|  | **Percentage (Enrichment)**  **Proteins** | **Percentage (Enrichment)**  **Proteins** | **Percentage (Enrichment)**  **Proteins** | **Percentage (Enrichment)**  **Proteins** |
| GO:0006979  response to oxidative stress | 6.32% (2.91e-02) | 2.17% (7.29e-01) | 8.03% (4.42e-04) | 0.51% (9.97e-01) |
|  | Ydl124w, Mcr1, Tma19, Sod1, Gad1, Hsp12 | Glr1, Ypr1, Trx1, Act1, Hsp104 | Grx4, Ydl124w, Gre3, Mcr1, Trx2, Gnd1, Tma19, Sod1, Gad1, Hsp12, Sod2 | Trx1 |
| GO:0016265  death | 8.42% (1.43e-05) | 0.87% (8.62e-01) | 5.84% (6.29e-04) | 0.51% (9.69e-01) |
|  | Atp3, Por1, Atp1, Atp2, Qcr2, Pet9, Cor1, Atp5 | Tdh2, Oye2 | Por1, Tdh3, Atp1, Atp2, Qcr2, Pet9, Mir1, Atp5 | Oye2 |

“Percentage”: the percentage of significantly changed proteins involved in each GO category.

“Enrichment”: the enrichment of significantly changed proteins in each GO category (hypergeometric distribution).
